# Who Can Truly Skip the Sentinel Node? A Paired Two-Stage Virtual Tumor-Board Study Among Patients Meeting the 2025 ASCO Clinicopathologic Criteria

**DOI:** 10.3390/diagnostics16213636

**Published:** 2026-11-04

**Authors:** Onur Can Güler, Ergun Yuksel, Veysel Cem Ozcan, Recep Dokuyucu

**Affiliations:** 1 Department of Surgical Oncology, Gülhane Medical Faculty, University of Health Sciences, Ankara Oncology Training and Research Hospital, 06200 Ankara, Türkiye; onurcguler@gmail.com; 2 Department of General Surgery, Gülhane Medical Faculty, University of Health Sciences, Ankara Oncology Training and Research Hospital, 06200 Ankara, Türkiye; 3 Department of Surgical Oncology, Antalya City Hospital, 07080 Antalya, Türkiye; cemzcan@gmail.com; 4 Department of Physiology, Medical Specialty Training Center (TUSMER), 06200 Ankara, Türkiye; drecepfatih@gmail.com

**Keywords:** breast cancer, sentinel lymph-node biopsy, axillary surgery, treatment de-escalation, multidisciplinary tumor board, radiotherapy, CDK4/6 inhibitor, decision-impact study

## Abstract

**Background/Objectives:** Sentinel lymph-node biopsy (SLNB) may be omitted in selected patients with clinically node-negative, early-stage breast cancer; however, the extent to which nodal information still influences contemporary adjuvant-treatment planning remains uncertain. This study aimed to determine whether disclosure of SLNB pathology changed multidisciplinary systemic-treatment and/or radiotherapy recommendations among patients fulfilling study-defined criteria derived from the 2025 American Society of Clinical Oncology omission pathway. **Methods:** In this single-center, retrospective, record-based paired decision-impact study, routine clinical records of consecutive postmenopausal women aged ≥ 50 years who underwent breast-conserving surgery and SLNB between 1 January 2015 and 30 June 2026 were reviewed after closure of the prespecified clinical-record window. Each anonymized case was assessed by a six-member virtual tumor board in two stages: first with SLNB pathology concealed and subsequently, after a six-week washout period, with nodal findings disclosed. The primary outcome was a definitive change in chemotherapy, CDK4/6-inhibitor, and/or radiotherapy recommendation. Paired transitions, interobserver agreement, decision confidence, and pre-SLNB factors associated with management-changing information were evaluated. **Results:** Among 412 eligible patients, occult sentinel-node deposits were identified in 46 (11.2%; 95% confidence interval [CI], 8.5–14.6%), including isolated tumor cells in seven, micrometastases in 13, and macrometastases in 26. Nodal disclosure produced at least one definitive management change in 28 patients (6.8%; 95% CI, 4.7–9.6%), corresponding to 14.7 SLNB procedures per management-changing recommendation. Twenty-six changes represented treatment escalation. Radiotherapy recommendations changed in 22 patients, CDK4/6-inhibitor recommendations in 13, and chemotherapy recommendations in seven, with overlap between domains. The expanded decision-impact endpoint was reached in 43 patients (10.4%; 95% CI, 7.8–13.8%). Management changed in 0.5% of patients with pN0 disease, none with isolated tumor cells, 46.2% with micrometastases, and 76.9% with macrometastases (*p* < 0.001). Increasing clinical tumor size was associated with management-changing information (adjusted odds ratio per 5 mm increase, 1.58; 95% CI, 1.07–2.37; *p* = 0.023). **Conclusions:** SLNB pathology left definitive adjuvant-treatment recommendations unchanged in more than 90% of selected patients but altered management in approximately one in 15, predominantly through radiotherapy-field selection and CDK4/6-inhibitor consideration. These findings support selective, multidisciplinary implementation of SLNB omission while emphasizing that decision changes do not necessarily represent proven oncological benefit.

## 1. Introduction

Axillary nodal status has traditionally been central to staging, prognostic assessment, and adjuvant-treatment planning in patients with clinically node-negative early breast cancer. Sentinel lymph node biopsy (SLNB) replaced axillary lymph-node dissection as the standard axillary staging procedure because it provides reliable pathological information with substantially lower morbidity. Nevertheless, SLNB is not a morbidity-free intervention. It may cause pain, sensory disturbance, arm swelling, reduced shoulder mobility, and lymphedema, in addition to increasing operative time and resource use. Patient-reported outcomes from the INSEMA trial demonstrated significantly greater and clinically relevant arm symptoms after SLNB than after omission of axillary surgery, with differences in pain, swelling, and mobility persisting throughout 18 months of postoperative follow-up [1,2]. As systemic treatment increasingly relies on tumor biology and genomic risk, the informational value of pathological nodal staging must therefore be weighed against even the relatively modest harms of SLNB.

Randomized evidence has substantially strengthened the case for omitting SLNB in carefully selected patients. In the SOUND trial, women with breast tumors measuring no more than 2 cm and negative preoperative axillary ultrasonography were assigned to SLNB or no axillary surgery. Five-year distant disease-free survival was 97.7% after SLNB and 98.0% without axillary surgery, establishing noninferiority, while postoperative systemic treatment and radiotherapy recommendations were similar between the groups [3]. The larger INSEMA trial subsequently evaluated patients with clinically node-negative T1–T2 invasive breast cancer undergoing breast-conserving therapy. After a median follow-up of approximately 6 years, omission of axillary surgery was noninferior to SLNB for invasive disease-free survival, with estimated 5-year rates of 91.9% and 91.7%, respectively [4]. Although axillary recurrence was numerically more frequent without axillary surgery (1.0% vs. 0.3%), patients in the omission group experienced less lymphedema, better arm mobility, and less pain during arm or shoulder movement [4]. These trials established the oncological safety of omission within selected populations, but they did not imply that SLNB had become dispensable for every clinically node-negative patient.

Reflecting these findings, the 2025 American Society of Clinical Oncology (ASCO) guideline update stated that SLNB may be omitted in selected patients who are postmenopausal and at least 50 years old; have a unifocal invasive ductal carcinoma measuring 2 cm or less, Nottingham grade 1–2, and hormone receptor-positive/HER2-negative disease; have negative preoperative axillary ultrasonography or a benign, concordant biopsy of a single suspicious node; intend to receive adjuvant endocrine therapy; and undergo upfront breast-conserving surgery. For patients aged < 65 years, the omission pathway additionally requires whole-breast irradiation after surgery [1]. Importantly, the recommendation is conditional on the expectation that detecting sentinel-node metastasis would not change systemic treatment or radiotherapy recommendations. The guideline further states that radiotherapy decisions, genomic testing, and subsequent systemic treatment decisions should not be altered solely because SLNB has been omitted in otherwise appropriate candidates [1]. Thus, anatomical and biological eligibility alone may not be sufficient; the individual value of SLNB ultimately depends on whether its result provides management-changing information.

The decision value of nodal information has become increasingly complex in the era of genomic assays and biology-directed therapy. RxPONDER demonstrated that postmenopausal patients with hormone receptor-positive/HER2-negative breast cancer, one to three positive lymph nodes, and a 21-gene recurrence score of 25 or lower did not derive a significant benefit from adjuvant chemotherapy, whereas premenopausal patients benefited from chemotherapy across recurrence-score categories [5]. These findings reduce the likelihood that occult low-volume nodal disease will alter chemotherapy recommendations in the population targeted by the 2025 ASCO omission criteria. Conversely, pathological nodal status may still modify anatomical stage, influence the choice between partial- and whole-breast irradiation or regional nodal irradiation, and determine eligibility for selected systemic intensification strategies, including adjuvant CDK4/6 inhibition [6]. Consequently, an SLNB result may have limited value for conventional chemotherapy selection yet remain relevant to other components of the contemporary postoperative treatment plan.

Recent observational studies confirm that this potential decision impact is small but not negligible. In a National Cancer Database analysis of 119,312 patients with cT1N0 hormone receptor-positive breast cancer, it was estimated that the absence of nodal pathology could affect at least one adjuvant decision in 7.9% of postmenopausal patients; this proportion decreased to 2.5% when ribociclib eligibility was excluded [7]. In a multicenter cohort of 867 patients meeting selected INSEMA-based criteria, occult nodal metastases were found in 14.3%, and nodal positivity was associated with potentially relevant treatment changes in 11.6% [8]. More recently, among 268 patients aged 50–70 years selected according to an ASCO-like phenotype, it was reported that SLNB findings potentially affected treatment recommendations in only 4.1%; most changes involved a shift from partial- to whole-breast irradiation, and recommendations remained unchanged in more than 95% of patients [9]. These estimates vary according to patient selection, genomic-testing availability, treatment guidelines, and institutional practice. Moreover, they have generally been derived from guideline algorithms or recorded treatment receipt rather than controlled, paired multidisciplinary assessments of the same patient with and without nodal information.

A recent single-center study provided proof of concept for such a paired approach [10]. Among 162 patients with clinically and sonographically node-negative pT1 breast cancer, the investigators presented the 25 node-positive cases to a simulated multidisciplinary team first with nodal status concealed and subsequently with complete pathological information. Disclosure of nodal status increased chemotherapy recommendations; however, the paired analysis was restricted to node-positive patients and evaluated only chemotherapy and genomic-testing decisions [10]. Therefore, the frequency with which SLNB changes the complete multidisciplinary treatment plan across the entire population meeting the 2025 ASCO omission criteria—including node-negative patients—remains insufficiently defined. The effects on genomic testing, cytotoxic chemotherapy, CDK4/6 inhibition, partial versus whole-breast or regional nodal irradiation, and further axillary management require a more comprehensive, denominator-based assessment.

Accordingly, this paired, two-stage virtual tumor-board study aimed to determine whether disclosure of sentinel lymph node pathology altered multidisciplinary adjuvant-treatment recommendations among patients who underwent breast-conserving surgery and SLNB but retrospectively met the objective clinicopathological and imaging criteria underpinning the 2025 ASCO recommendation for SLNB omission.

## 2. Materials and Methods

### 2.1. Study Design and Setting

This single-center, retrospective, record-based paired decision-impact study was conducted in the Department of General Surgery, University of Health Sciences Ankara Dr. Abdurrahman Yurtaslan Oncology Training and Research Hospital, Ankara, Türkiye. Routine clinical records of consecutive patients who underwent surgery for primary invasive breast cancer between 1 January 2015 and 30 June 2026 were retrospectively reviewed. This interval represented the prespecified clinical-record window rather than a period of prospective patient enrollment. This study evaluated the incremental contribution of sentinel lymph node biopsy (SLNB) pathology to contemporary multidisciplinary treatment recommendations by comparing decisions made for the same patient before and after disclosure of nodal information. The study protocol was approved by the Abdurrahman Yurtaslan Ankara Oncology Training and Research Hospital Ethics Committee on 23 May 2026 (Approval No. E-2026-5) and covered the review of routinely generated clinical records through the prespecified administrative cutoff of 30 June 2026. No prospective patient enrollment, research-related intervention, or alteration of routine clinical care occurred. The database was closed after 30 June 2026, and research-specific eligibility verification, data extraction, de-identification, preparation of virtual case summaries, virtual tumor-board assessments, and statistical analyses were subsequently performed under the approved protocol. This study was conducted in accordance with the Declaration of Helsinki and applicable institutional data-protection regulations. The virtual tumor-board assessments were performed solely for research purposes after routine clinical management and did not influence the treatment received by any patient.

After closure of the prespecified clinical-record window, potentially eligible cases were identified retrospectively by cross-referencing institutional surgical records, pathology databases, radiology reports, and electronic medical records. Eligibility was determined using information available before SLNB and according to a study-specific algorithm based on Recommendation 1.1 of the 2025 American Society of Clinical Oncology (ASCO) guideline [1].

Patients were eligible for the primary cohort if they met all the following criteria: female sex; age ≥ 50 years and documented postmenopausal status at diagnosis; newly diagnosed, unilateral, and unifocal invasive ductal carcinoma/invasive breast carcinoma of no special type; clinical tumor size ≤ 2 cm; Nottingham grade 1 or 2 on preoperative core-needle biopsy; hormone receptor-positive and HER2-negative disease; clinically and sonographically node-negative axilla; upfront breast-conserving surgery; successful SLNB with an available definitive pathology report; and eligibility for endocrine therapy. Eligibility for endocrine therapy was determined from the preoperative treatment plan and the absence of a documented contraindication; actual postoperative treatment receipt was not used to establish eligibility. A negative axillary evaluation was defined as the absence of morphologically suspicious lymph nodes on preoperative ultrasonography or the presence of only one suspicious node with a benign and imaging-concordant needle-biopsy result. For women aged 50–59 years, postmenopausal status had to be explicitly documented; age alone was not considered sufficient. For patients aged < 65 years, eligibility additionally required a documented preoperative intention to deliver whole-breast irradiation after breast-conserving surgery, consistent with ASCO Recommendation 1.1 [1]. Preoperative radiotherapy intent was abstracted from multidisciplinary or radiation-oncology records and categorized as whole-breast irradiation planned, another radiotherapy approach planned, no radiotherapy planned, or undocumented. Actual postoperative radiotherapy and the Stage 1 or Stage 2 virtual tumor-board recommendations were not used to determine eligibility.

Patients were excluded if they had multifocal or multicentric disease, invasive lobular carcinoma or another nonductal invasive histology, clinical or biopsy-confirmed axillary nodal involvement, a clinical tumor size > 2 cm, grade 3 disease on diagnostic biopsy, HER2-positive or triple-negative disease, distant metastasis, neoadjuvant systemic therapy, mastectomy, bilateral breast cancer, previous ipsilateral invasive breast cancer, previous ipsilateral axillary surgery or radiotherapy, inflammatory breast cancer, or insufficient clinical, imaging, pathological, or SLNB data. Patients with pure ductal carcinoma in situ or microinvasive disease without a clearly documented invasive component were also excluded.

Eligibility for the primary analysis was determined using information available before surgery. Patients who fulfilled the preoperative eligibility criteria therefore remained in the primary cohort even when definitive breast pathology revealed a tumor size > 20 mm, grade 3 disease, multifocality, or a previously unrecognized nonductal invasive component. These postoperative findings were recorded as separate, non-mutually exclusive features. For the strictly postoperatively confirmed low-risk sensitivity analysis, patients were required to have unifocal invasive ductal carcinoma/invasive breast carcinoma of no special type measuring ≤20 mm, Nottingham grade 1–2, and hormone receptor-positive/HER2-negative disease on definitive pathology. Patients with more than one disqualifying postoperative feature were excluded only once. Sentinel-node status was not used to select this subgroup, and lymphovascular invasion alone was not an exclusion criterion. Essential eligibility variables were verified independently by two investigators, with disagreements resolved through review by a senior breast surgeon.

### 2.2. Clinicopathological Data Collection

The following variables were extracted: age at diagnosis, menopausal status, body mass index, comorbidities, American Society of Anesthesiologists physical status, family history of breast cancer, clinical tumor size, imaging modality used for tumor measurement, axillary ultrasonography findings, method and result of any axillary needle biopsy, core-biopsy histology and grade, estrogen receptor (ER), progesterone receptor (PR), HER2, and Ki-67 status, type of breast-conserving procedure, definitive pathological tumor size and grade, lymphovascular invasion, multifocality, margin status, and genomic assay results when available. Genomic-result availability was defined by the presence of a documented, interpretable assay report in the records available for case review, rather than by a recommendation or order for testing alone. The assay platform, reported numerical score and/or assay-specific risk category, and report date were recorded when available. Patients without an interpretable report were classified as having no available genomic result; absence of a result was not interpreted as a low-risk result. ER and PR were considered positive when ≥1% of invasive tumor-cell nuclei demonstrated immunohistochemical staining. ER expression of 1–10% was additionally recorded as ER-low positive [11]. HER2-negative disease was defined as immunohistochemistry 0 or 1+, or immunohistochemistry 2+ without amplification on in situ hybridization, in accordance with ASCO/College of American Pathologists recommendations [12].

SLNB variables included the mapping technique, number of sentinel nodes removed, number of involved nodes, size of the largest metastatic deposit, presence of isolated tumor cells, micrometastasis or macrometastasis, extranodal extension, and whether subsequent axillary treatment was performed. Isolated tumor cells were defined as deposits ≤0.2 mm or ≤200 cells, micrometastases as deposits >0.2 mm but ≤2 mm, and macrometastases as deposits >2 mm. For the primary description of occult nodal disease, pN0(i+), pN1mi, and macrometastatic disease were reported separately rather than combined into a single node-positive category. Original postoperative oncology recommendations, treatments received, subsequent axillary procedures, recurrence data, and survival outcomes were collected only for exploratory analyses.

### 2.3. Preparation of Virtual Case Summaries

A standardized and anonymized electronic case summary was created for each eligible patient. The summary contained age, menopausal status, relevant comorbidities, preoperative breast and axillary imaging, core-biopsy findings, surgical procedure, and all definitive breast pathology findings that would have remained available if SLNB had been omitted. Pathological nodal stage, node-dependent anatomical stage, number of sentinel nodes examined or involved, metastasis size, extranodal extension, completion axillary surgery, actual postoperative treatment, treatment year, recurrence, and survival outcomes were removed from the first-stage summary. In the node-concealed stage, the axilla was described as clinically and sonographically node-negative, with pathological nodal status unavailable (pNx); patients were not assumed to be pathologically node-negative. Existing genomic-assay results were concealed at both stages of the primary assessment because the decision to request testing was itself an outcome. This information restriction could also leave chemotherapy recommendations conditional on genomic testing. Accordingly, “await genomic testing” was retained as a distinct recommendation category and was not treated as a definitive recommendation against chemotherapy.

A paired sensitivity assessment evaluated treatment recommendations when each existing, interpretable genomic result was displayed identically at both the node-concealed and node-disclosed stages. For patients without an available result, genomic information remained unavailable at both stages; no result was imputed. All other case information and the treatment-decision criteria were held constant. This analysis therefore evaluated the incremental effect of nodal disclosure in a setting where existing genomic information was already available.

### 2.4. Virtual Tumor-Board Procedure

The virtual tumor board included voting members from breast/general surgery, medical oncology, and radiation oncology. Breast radiology and pathology specialists were available to verify imaging and pathological variables but did not provide information that was masked by the protocol. Before case assessment, a written decision manual and standardized response form were finalized. Pilot cases not included in the study cohort were used to familiarize panel members with the assessment procedure. The treatment-specific decision rules relevant to chemotherapy, genomic testing, CDK4/6 inhibition, and radiotherapy are provided in Supplementary Text S1.

During the first stage, all cases were evaluated with SLNB pathology concealed. Panel members initially recorded their recommendations independently and rated their confidence on a 5-point Likert scale. The multidisciplinary consensus recommendation was subsequently determined after discussion. The recommendations were systematically categorized across several clinical domains to ensure clarity and consistency. Within the domain of genomic assays, guidance was recorded as either not required, recommended, or indeterminate. For cytotoxic chemotherapy, recommendations were classified as recommended, not recommended, or conditional upon genomic testing results. In the case of endocrine therapy, treatment was designated as standard duration, extended treatment considered, or contraindicated. Regarding adjuvant CDK4/6 inhibition, the options included recommended, not recommended, or indeterminate. For radiotherapy, the categories encompassed omission, partial-breast irradiation, whole-breast irradiation, or whole-breast irradiation with regional nodal irradiation. Finally, in terms of further axillary management, the recorded strategies were none, axillary radiotherapy, completion axillary lymph-node dissection, or additional evaluation required.

First-stage individual and consensus decisions were electronically locked before nodal information was released. After a six-week washout period, cases were assigned new study codes and presented in a newly randomized order. Panel members did not have access to their first-stage responses.

During the second stage, the same nonnodal information was provided together with the SLNB findings, including the number of sentinel nodes examined and involved, the category and size of the largest metastatic deposit, and extranodal extension. Results of any subsequent completion axillary dissection remained concealed because they constituted downstream information rather than information generated directly by SLNB. Independent and consensus recommendations were again recorded using the same structured response form.

All cases were reassessed according to treatment standards current at the time of the virtual review, regardless of the calendar year in which the original surgery was performed. The documented preoperative intention to deliver whole-breast irradiation in patients younger than 65 years was used to establish cohort eligibility and did not constrain the subsequent virtual tumor-board recommendations. The panel was permitted to reconsider the indication for breast irradiation and the appropriate treatment volume using the information available at each assessment stage. A recommendation for omission or partial-breast irradiation therefore represented reassessment of the original treatment plan rather than retrospective modification of eligibility. For patients aged 50–64 years receiving either recommendation, the documented rationale was reviewed separately. Where no rationale was recorded, this was reported explicitly rather than inferred from clinicopathological features. Decision making was informed by the 2025 ASCO SLNB guideline, contemporary genomic-assay guidance, evidence concerning adjuvant chemotherapy and CDK4/6 inhibition, and current partial-breast and regional radiotherapy recommendations [1,13,14]. Radiotherapy recommendations distinguished between the indication for breast irradiation, the choice of partial- versus whole-breast irradiation, and the addition of regional nodal irradiation (RNI). Disclosure of sentinel-node involvement did not automatically mandate RNI. Nodal findings were interpreted according to the number of involved sentinel nodes, the size of the largest metastatic deposit, and the presence and extent of extranodal extension, together with definitive breast tumor size, histological grade, lymphovascular invasion, age, and comorbidity. Definitive breast pathology and other nonnodal characteristics were identical in both assessment stages; these features therefore provided the clinical context in which the newly disclosed nodal information was interpreted.

Isolated tumor cells were not considered an indication for nodal irradiation. Micrometastatic disease alone was not treated as a mandatory indication for RNI; whole-breast irradiation without elective nodal irradiation remained an option when the remaining clinicopathological features were favorable. For micrometastatic disease accompanied by additional adverse features, RNI was considered through individualized multidisciplinary assessment. Macrometastatic disease prompted assessment of the potential value of RNI, with greater nodal burden, extranodal extension, lymphovascular invasion, grade 3 disease, and an unexpectedly larger definitive tumor size favoring consideration of treatment escalation. One or two involved sentinel nodes did not, in isolation, require RNI in every patient [1,15].

The suitability of partial-breast irradiation was assessed separately. Detection of micrometastatic or macrometastatic nodal disease could support a transition from partial- to whole-breast irradiation because standard partial-breast irradiation recommendations do not include patients with positive lymph nodes. Such a transition was distinguished from a recommendation to add RNI [13].

Chemotherapy recommendations were assessed separately from genomic-test ordering. Sentinel-node involvement was not treated as an automatic indication for chemotherapy in this postmenopausal, hormone receptor-positive/HER2-negative population. The board considered nodal findings together with definitive tumor characteristics and the potential contribution of genomic testing [5,14]. When genomic testing was considered necessary before a definitive chemotherapy decision, the recommendation remained conditional. Any transition from a conditional recommendation to a definitive recommendation for chemotherapy after nodal disclosure required a documented case-specific rationale. These transitions were reported separately from transitions from “chemotherapy not recommended” to “chemotherapy recommended.” Genomic-assay results concealed during the primary assessment were not used to justify the original stage-specific decisions.

Eligibility for adjuvant CDK4/6 inhibition was assessed separately for abemaciclib and ribociclib using explicitly defined clinicopathological criteria. For abemaciclib, the study definition followed the monarchE cohort 1 framework: hormone receptor-positive/HER2-negative, node-positive disease with either ≥4 positive axillary lymph nodes or 1–3 positive axillary lymph nodes accompanied by histological grade 3 disease and/or a primary tumor measuring ≥50 mm. Ki-67 ≥ 20% alone was not considered sufficient under this cohort 1-based definition [16].

For ribociclib, eligibility was assessed using the NATALEE framework based on AJCC eighth-edition anatomical stage rather than prognostic stage. Eligible categories included stage IIA disease with N1 involvement, stage IIB disease, and stage III disease. For stage IIA node-negative disease, eligibility required grade 3 disease or grade 2 disease accompanied by Ki-67 ≥ 20% or a qualifying high genomic-risk result, such as an Oncotype DX recurrence score ≥ 26 [6,17]. Isolated tumor cells and pT1pN1mi disease were not considered sufficient, by themselves, to establish eligibility through the node-positive pathway [18].

These criteria were applied using only the information available at each assessment stage. During Stage 1, pathological nodal status remained pNx; neither pathological node negativity nor node positivity was assumed. During Stage 2, eligibility was reassessed after disclosure of sentinel-node findings, while all nonnodal information remained unchanged. Findings from subsequent completion axillary dissection were not used to establish eligibility. Existing genomic-assay results remained concealed in the primary assessment; an eligibility criterion requiring an unavailable genomic result was therefore not assumed to have been met.

Formal eligibility was distinguished from the final treatment recommendation. Panelists considered the applicable drug-specific criteria alongside comorbidity, anticipated toxicity, and expected absolute benefit before recording their independent recommendations and reaching multidisciplinary consensus. Eligibility alone did not automatically generate a recommendation for treatment. Recommendations remained classified as recommended, not recommended, or indeterminate.

Panel members were blinded to the original multidisciplinary recommendations, treatment received, patient outcomes, and the overall frequency of nodal positivity within the cohort. If a panel member recognized a patient or had participated directly in the original treatment decision, this was recorded, and the member did not contribute to that patient’s independent rating. Unresolved consensus disagreements were adjudicated by a senior specialist who had not participated in the initial vote. Age-specific radiotherapy recommendations, complete paired transitions, the additional sensitivity analysis, and patient-level explanations for recommendations outside the whole-breast irradiation scenario are presented in Supplementary Table S3 (panels A–D).

### 2.5. Study Outcomes

The patient was the unit of analysis. The conservative primary endpoint was the proportion of patients in the entire eligible cohort in whom disclosure of SLNB pathology produced at least one change between definitive Stage 1 and Stage 2 treatment recommendations. For chemotherapy and CDK4/6-inhibitor therapy, a qualifying change required a transition from “do not recommend” to “recommend,” or the reverse, with a definitive recommendation at both stages. For radiotherapy, a change among omission, partial-breast irradiation, whole-breast irradiation, and whole-breast irradiation with regional nodal irradiation qualified as a primary event. Each patient was counted once, irrespective of the number of qualifying treatment domains.

Transitions from “await genomic testing” to either “recommend chemotherapy” or “do not recommend chemotherapy” were classified as resolution of a conditional recommendation and were analyzed as secondary outcomes. Similarly, transitions from an indeterminate CDK4/6-inhibitor recommendation to a definitive recommendation for or against treatment were classified as secondary outcomes. These transitions did not independently constitute primary endpoint events. A patient with such a transition could nevertheless meet the primary endpoint through a qualifying definitive treatment change in another domain.

The expanded decision-impact endpoint included patients with a primary endpoint event, any change in genomic-testing recommendation category among “not required,” “recommended,” and “indeterminate,” or resolution of a conditional chemotherapy or indeterminate CDK4/6-inhibitor recommendation. Patients meeting more than one criterion were counted once. Development of a new conditional or indeterminate recommendation, or a change in additional axillary management alone, did not independently qualify for the expanded endpoint. Changes limited to wording, treatment sequencing, dose or fractionation, or confidence level did not qualify for either endpoint.

Key secondary outcomes included initiation or cancellation of genomic testing; resolution of an indeterminate or conditional recommendation; recommendation for additional axillary treatment; domain-specific decision-change rates; direction of change as escalation or de-escalation; number of affected treatment domains per patient; changes in decision confidence; and interobserver agreement before consensus. Additional outcomes included the prevalence and burden of occult nodal metastasis, the number of SLNB procedures required to identify one patient with management-changing nodal information, and clinicopathological characteristics associated with a decision-changing SLNB result. Detailed transition rules, the complete genomic-testing transition matrix, and the mutually exclusive composition of the expanded endpoint are provided in Supplementary Table S2A–C, respectively.

### 2.6. Statistical Analysis

Analyses were performed using IBM SPSS Statistics version 27.0 (IBM Corp., Armonk, NY, USA) and R version 4.3.0 (R Foundation for Statistical Computing, Vienna, Austria). Continuous variables were summarized as means ± standard deviations or medians (interquartile ranges), as appropriate, and categorical variables as numbers and percentages. Exploratory baseline comparisons used independent-samples *t* tests or Mann–Whitney U tests for continuous variables and Pearson’s chi-square or Fisher’s exact tests for categorical variables. Each patient was counted once within each endpoint, irrespective of the number of affected treatment domains. Primary and expanded decision-impact rates and occult nodal-disease prevalence were reported with two-sided 95% Wilson confidence intervals (CIs). The number of SLNB procedures per management-changing recommendation was calculated as the reciprocal of the primary event proportion, with confidence limits obtained by inverting its Wilson limits.

Exact McNemar tests for chemotherapy and CDK4/6-inhibitor recommendations included only patients with definitive recommendations at both stages. Resolution of conditional or indeterminate recommendations was analyzed separately as a secondary outcome. For genomic testing, “recommended” was compared with “not recommended or indeterminate” solely for the binary test; the complete three-category transition matrix was reported. Radiotherapy transitions were summarized in paired matrices, compared using the Stuart–Maxwell test, and classified as escalation or de-escalation according to increasing or decreasing treatment extent. Associations between nodal-burden categories and the primary endpoint were evaluated using the Fisher–Freeman–Halton exact test.

Agreement among the six voting panelists was assessed separately at each stage using mean pairwise percentage agreement and Fleiss’ κ with 95% CIs; κ was reported as not estimable when recommendation variability was insufficient. Consensus without adjudication was summarized descriptively. Patient-level confidence scores were averaged across panelists and compared between stages using the Wilcoxon signed-rank test, with the Hodges–Lehmann paired location shift and its 95% CI reported.

Exploratory univariable and multivariable Firth logistic regression evaluated age per 10-year increase, clinical tumor size per 5 mm increase, and core-biopsy grade 2 versus 1. Results were expressed as odds ratios with profile penalized-likelihood 95% CIs and penalized likelihood-ratio *p* values. No automated variable selection was used. Ki-67 per 5-percentage-point increase was added in a separate complete-case sensitivity model. No missing values were imputed.

Sensitivity analyses examined the strictly postoperatively confirmed low-risk subgroup, grouping pN0(i+) with node-negative disease, and availability of existing genomic results. In the genomic-information assessment, interpretable results were displayed identically at both nodal-information stages; unavailable results remained unavailable. The same primary endpoint rules were applied, and chemotherapy transitions and patient-level endpoint concordance were reported. Age-specific radiotherapy recommendations and transitions were reported. An additional reviewer-requested analysis excluded patients aged 50–64 years recommended radiotherapy omission or partial-breast irradiation at either stage, recalculating both numerator and denominator with each patient counted once. Decision-change rates for 2015–2020 versus 2021–2026 were compared using Fisher’s exact test. All tests were two-sided, with a nominal significance threshold of *p* < 0.05. Secondary and subgroup analyses were exploratory, without multiplicity adjustment, and their *p* values were interpreted descriptively.

## 3. Results

Following closure of the clinical-record window, the records of 3684 consecutive patients who underwent surgery for primary invasive breast cancer between 1 January 2015 and 30 June 2026 were retrospectively reviewed for eligibility. After sequential exclusion of 3272 patients who did not satisfy the predefined eligibility criteria, 412 patients constituted the primary study cohort and completed both stages of the virtual tumor-board assessment. A total of 43 patients (10.4%) had at least one unexpected postoperative finding, including pathological tumor size > 2 cm, grade 3 disease, multifocality, or a nonductal component. Consequently, 369 patients constituted the strictly postoperatively confirmed low-risk cohort (Figure 1).

The mean age was 65.0 ± 7.7 years, and 215 patients (52.2%) were aged ≥ 65 years. The median clinical tumor size was 14 mm (interquartile range [IQR], 10–17 mm); 261 tumors (63.3%) were grade 2 on core biopsy; and the median Ki-67 proliferation index was 14% (IQR, 8–21%). Compared with patients without a sentinel-node tumor deposit, patients with an occult nodal deposit had larger clinical tumors, were more frequently diagnosed with grade 2 disease, and had higher Ki-67 values. They also had larger definitive pathological tumor sizes and a higher prevalence of lymphovascular invasion. Unexpected postoperative pathological features comprised invasive tumor size > 20 mm, Nottingham grade 3, multifocality, and a previously unrecognized nonductal invasive component. Age, body mass index, axillary ultrasonography category, and type of breast-conserving surgery did not differ significantly between groups (Table 1).

The median number of sentinel nodes removed was 2 (IQR, 1–3). Any occult sentinel-node tumor deposit was identified in 46 patients (11.2%; 95% confidence interval [CI], 8.5–14.6%). Seven patients (1.7%) had isolated tumor cells, 13 (3.2%) had micrometastases, and 26 (6.3%) had macrometastatic disease. Thus, micrometastatic or macrometastatic nodal involvement was present in 39 patients (9.5%). Among patients with a nodal deposit, 38 (82.6%) had one involved sentinel node, six (13.0%) had two involved nodes, and two (4.3%) had three involved nodes. Extranodal extension was identified in six patients (13.0% of patients with a nodal deposit) (Table 2).

Disclosure of SLNB pathology produced at least one clinically meaningful change in the definitive systemic treatment or radiotherapy recommendation in 28 of 412 patients (6.8%; 95% CI, 4.7–9.6%). The corresponding number of SLNB procedures per management-changing recommendation required to obtain one management-changing result was 14.7 (95% CI, 10.4–21.1). Of the 28 affected patients, 26 (92.9%) underwent treatment escalation and two (7.1%) underwent de-escalation. Seventeen patients had a change in one treatment domain, eight had changes in two domains, and three had simultaneous changes in chemotherapy, CDK4/6-inhibitor, and radiotherapy recommendations. Radiotherapy recommendations changed in 22 patients, CDK4/6-inhibitor recommendations in 13, and definitive chemotherapy recommendations in seven. Recommendations for adjuvant CDK4/6 inhibition increased from two patients (0.5%) during the node-concealed assessment to 15 patients (3.6%) after nodal disclosure, comprising 13 newly recommended treatments and no withdrawn recommendations. Because some patients had changes in more than one domain, these domain-specific counts were not mutually exclusive. When changes in genomic-testing category and resolution of conditional or indeterminate recommendations were additionally considered, the expanded decision-impact endpoint was reached in 43 patients (10.4%; 95% CI, 7.8–13.8%). This comprised the 28 patients meeting the primary endpoint and 15 additional patients with secondary decision changes only; patients with overlapping changes were counted once (Table 3, Figure 2, and Supplementary Table S2C).

The genomic-testing recommendation category changed in 32 of 412 patients (7.8%). These changes comprised 15 transitions into the “recommended” category, 10 transitions out of that category, and seven transitions between “not required” and “indeterminate.” The number of patients for whom testing was recommended therefore increased from 96 at Stage 1 to 101 at Stage 2, with 86 patients receiving a recommendation for testing at both stages. Thus, the 32 category changes should not be interpreted as 32 newly recommended assays. The complete transition matrix is presented in Supplementary Table S2B.

Radiotherapy recommendations changed in 22 of 412 patients (5.3%), comprising 20 escalations and two de-escalations. RNI was newly recommended in 12 patients (2.9% of the entire cohort): 11 transitioned from whole-breast irradiation to whole-breast irradiation plus RNI, and one transitioned from partial-breast irradiation to whole-breast irradiation plus RNI. The remaining eight escalations involved breast irradiation without the addition of RNI: six patients transitioned from partial- to whole-breast irradiation, and two transitioned from radiotherapy omission to whole-breast irradiation. Both de-escalations occurred in patients with pathologically negative sentinel nodes, with one transition from whole- to partial-breast irradiation and one from whole-breast irradiation to omission. Thus, the 22 radiotherapy changes included 12 additions of RNI and ten changes involving breast irradiation or its omission without adding RNI. The overall radiotherapy distribution differed between stages (Stuart–Maxwell χ^2^ = 15.49, 3 degrees of freedom, *p* = 0.001). The cohort included 197 patients aged 50–64 years and 215 patients aged ≥ 65 years. Age-specific radiotherapy recommendations at both assessment stages and the complete paired transition matrices are presented in Supplementary Table S3A,B. The clinicopathological features and recorded reasons for recommending radiotherapy omission or partial-breast irradiation in younger patients are detailed in Supplementary Table S3D. An additional sensitivity analysis excluded patients aged 50–64 years who received either recommendation at either assessment stage. The conservative primary endpoint was recalculated in the remaining cohort, with each patient counted once irrespective of overlapping treatment changes. The number of excluded patients, their contribution to the primary endpoint, and the resulting event count, denominator, proportion, and 95% Wilson confidence interval are reported in Supplementary Table S3C. Nodal disclosure resulted in a recommendation for active additional axillary treatment in ten patients (2.4%), including axillary radiotherapy in eight and completion axillary lymph-node dissection in two. Additional imaging or pathological evaluation before determining axillary treatment was recommended in three further patients (Table 4).

Because only 28 primary events occurred, the prespecified multivariable Firth model was restricted to age, clinical tumor size, and core-biopsy grade. Increasing clinical tumor size was associated with the outcome after adjustment with management-changing nodal information (adjusted odds ratio [OR] per 5 mm increase, 1.58; 95% CI, 1.07–2.37; *p* = 0.023). Neither age nor core-biopsy grade was significantly associated with the primary outcome after adjustment. Ki-67 was evaluated only in a complete-case sensitivity model comprising 366 patients and 25 primary events. Ki-67 was not independently associated with decision change after adjustment (adjusted OR per 5-percentage-point increase, 1.10; 95% CI, 0.96–1.26; *p* = 0.170). These analyses were considered exploratory (Table 5 and Figure 3).

Twelve patients received a new recommendation for RNI following disclosure of sentinel-node pathology. Their nodal disease category, number of involved sentinel nodes, metastatic deposit size, accompanying clinicopathological risk features, paired radiotherapy recommendations, and documented rationale for RNI are detailed in Supplementary Table S1, which summarizes treatment-recommendation changes in all 28 patients reaching the primary endpoint.

Clinically meaningful changes were strongly associated with the burden of sentinel-node disease. A treatment change occurred in two of 366 patients with pN0 disease (0.5%), none of seven with isolated tumor cells, six of 13 with micrometastatic disease (46.2%), and 20 of 26 with macrometastatic disease (76.9%; *p* < *0*.001). Overall, SLNB disclosure changed treatment in 26 of 46 patients with any nodal tumor deposit (56.5%) and in 26 of 39 patients with micrometastatic or macrometastatic disease (66.7%). Both changes among patients with pN0 disease represented radiotherapy de-escalation (Table 6).

Six voting specialists completed both virtual assessment stages. Interobserver agreement increased after nodal disclosure for genomic testing, chemotherapy, CDK4/6 inhibition, and radiotherapy, whereas percentage agreement for further axillary management decreased slightly from 96.8% to 95.6%. The largest increases in Fleiss’ κ were observed for CDK4/6-inhibitor recommendations and radiotherapy categories. Consensus without adjudication was achieved for 389 patients (94.4%) during the node-concealed stage and 400 (97.1%) during the node-disclosed stage. Median case-level decision confidence increased from 3.7 (IQR, 3.3–4.0) before nodal disclosure to 4.2 (IQR, 3.8–4.5) afterward. The Hodges–Lehmann median paired difference was 0.50 points (95% CI, 0.42–0.58; *p* < 0.001) (Table 7).

Among the 369 patients who retained all specified low-risk breast-pathology characteristics after definitive pathological assessment, the conservative primary endpoint occurred in 21 patients (5.7%; 95% CI, 3.8–8.5%). Grouping pN0(i+) with node-negative disease did not alter the nodal-burden subgroup findings; none of the seven patients with pN0(i+) disease experienced a primary treatment change. When available genomic assay results were disclosed identically at both stages, the primary endpoint was reached in 25 patients (6.1%), compared with 28 patients (6.8%) in the main analysis. Decision-change rates were also similar between patients treated during 2015–2020 and those treated during 2021–2026 (6.5% vs. 7.0%, respectively; *p* = 1.000).

## 4. Discussion

In this single-center, paired, two-stage virtual tumor-board study, disclosure of SLNB pathology changed at least one definitive systemic-treatment or radiotherapy recommendation in 28 of 412 patients (6.8%), whereas the recommendations remained unchanged in 93.2%. When genomic-test ordering and resolution of conditional or indeterminate recommendations were additionally considered, the expanded decision-impact rate was 10.4%. Most definitive changes represented treatment escalation and were concentrated in radiotherapy and CDK4/6-inhibitor recommendations, while definitive chemotherapy changes were uncommon. The informational yield of SLNB was therefore modest at the population level but substantial among patients with clinically relevant nodal disease: management changed in 66.7% of patients with micrometastatic or macrometastatic involvement, compared with 0.5% of those with pN0 disease and none of those with isolated tumor cells. The reciprocal estimate of 14.7 SLNB procedures per management-changing recommendation should be interpreted as a measure of informational yield rather than as a number needed to treat, because this study did not determine whether the altered recommendations improved recurrence, survival, or quality of life.

Wanis et al. estimated from a National Cancer Database cohort that the absence of nodal pathology could affect at least one adjuvant-treatment decision in 7.9% of postmenopausal patients with cT1N0 hormone receptor-positive breast cancer; this estimate decreased to 2.5% when ribociclib eligibility was excluded [7]. Tauber et al. identified occult nodal metastases in 14.3% of 867 patients selected using INSEMA-related criteria and estimated that nodal information could produce potentially relevant treatment changes in 11.6% [8]. Namoglu et al. reported a lower overall decision-impact rate of 4.1% among 268 women aged 50–70 years with an ASCO-like low-risk phenotype; nine of the 11 identified changes involved replacement of partial-breast irradiation with whole-breast irradiation [9]. Landry et al. used a paired multidisciplinary design and found that disclosure of nodal status increased chemotherapy recommendations from eight to 16 among 25 node-positive patients; however, that analysis was restricted to node-positive cases and included broader biological subtypes, making its rate unsuitable for direct denominator-based comparison with our study [10]. Pilewskie et al. found that SLNB findings affected chemotherapy appropriateness or the indication for axillary dissection in approximately 1% of postmenopausal patients, regional nodal irradiation in 3%, and partial-breast irradiation eligibility in 13% [19]. Our primary decision-impact rate lies within the range of these studies but reflects a different endpoint: an actual paired multidisciplinary recommendation rather than algorithmically defined treatment eligibility or treatment received. The distinction is important because meeting eligibility criteria for an intervention does not necessarily mean that the intervention would be recommended after individualized multidisciplinary assessment.

Randomized omission trials provide the oncological context for these findings. Gentilini et al. reported in the SOUND trial that micrometastatic or macrometastatic disease was found in 13.7% of patients assigned to SLNB, yet omission of axillary surgery was noninferior for 5-year distant disease-free survival, and the 5-year cumulative incidence of axillary recurrence was 0.4% in both groups [3]. Reimer et al. similarly demonstrated in INSEMA that omission of axillary surgery was noninferior for invasive disease-free survival, although axillary recurrence was numerically more frequent without axillary surgery than with SLNB (1.0% vs. 0.3%) [4]. The 9.5% prevalence of micrometastatic or macrometastatic disease in our cohort was slightly lower, plausibly because of the more restrictive selection of postmenopausal patients with tumors measuring ≤2 cm, grade 1–2 disease, hormone receptor-positive/HER2-negative biology, and negative axillary imaging. Importantly, SOUND and INSEMA evaluated the oncological safety of omitting axillary surgery, whereas the present study evaluated the incremental effect of nodal information on multidisciplinary decisions. Accordingly, the 6.8% decision-change rate must not be interpreted as the proportion of patients who would experience an adverse oncological outcome if SLNB were omitted.

The observed prevalence and distribution of occult nodal disease were consistent with contemporary real-world cohorts. Giannakou et al. reported SLN positivity in 12.2% of 312 SOUND-eligible patients, but only 0.4% had four or more involved nodes; among postmenopausal patients with a 21-gene recurrence score ≤ 25, chemotherapy recommendations were not associated with nodal status [20]. Campbell et al. found nodal involvement in 10.9% of 755 surgically staged patients and demonstrated a gradient according to clinical tumor size, from 5.2% for cT1b to 12.8% for cT1c tumors [21]. Jackson et al. similarly reported nodal positivity in 10.9% of 1194 patients with cT1N0, estrogen receptor-positive/HER2-negative, grade 1–2 disease, with only 0.4% having four or more positive nodes; nodal involvement increased with tumor size and decreased with increasing age [22]. These findings support the external plausibility of the 11.2% prevalence of any nodal deposit in our cohort. They also support the exploratory association between increasing clinical tumor size and management-changing nodal information observed in our Firth model. Nevertheless, the adjusted odds ratio of 1.58 per 5 mm increase was based on only 28 events, and the absence of independent associations with age, core-biopsy grade, or Ki-67 may reflect restricted variable ranges and limited statistical power. Clinical tumor size may therefore help enrich the probability of obtaining actionable nodal information, but it cannot yet be used as a validated selection rule.

Radiotherapy was the treatment domain most frequently affected by nodal disclosure, with changes occurring in 22 of the 28 patients who reached the primary endpoint. These changes comprised 12 new recommendations for RNI, eight escalations involving breast irradiation without adding RNI, and two de-escalations. The distinction between a transition from partial- to whole-breast irradiation and the addition of nodal irradiation is clinically relevant. Detection of nodal metastasis may make a patient unsuitable for partial-breast irradiation under current recommendations without establishing that RNI is necessary or beneficial in every such patient [1,13]. Pilewskie et al. similarly found that nodal information resulted in regional nodal irradiation in 3% of their entire cohort and rendered 13% of initially eligible patients unsuitable for partial-breast irradiation [19]. Namoglu et al. also identified the transition from partial- to whole-breast irradiation as the predominant consequence of SLNB information [9]. Behzadi et al. demonstrated dosimetrically that whole-breast irradiation delivers substantially greater incidental dose to the sentinel-node region than partial-breast irradiation, highlighting the need for caution when combining SLNB omission with highly restricted radiation fields [23]. Taylor et al. showed in an individual-patient meta-analysis of 14,324 women that contemporary regional nodal irradiation reduced breast-cancer recurrence and mortality, but the absolute benefit varied according to nodal burden and baseline recurrence risk [15]. This broader evidence does not establish that escalation to regional nodal irradiation benefits patients with the small-volume, imaging-occult nodal disease represented in our cohort. Thus, a management change in a virtual tumor board demonstrates the residual decision value of nodal information but does not itself prove a favorable risk–benefit ratio for treatment escalation.

Shaitelman et al. emphasized the importance of clinical, pathological, and treatment-related factors when selecting patients for partial-breast irradiation [13]. Park et al. further stated that radiotherapy decisions should not be altered solely because SLNB has been omitted in an otherwise appropriate candidate [1]. Reimer et al. evaluated SLNB omission within a breast-conserving treatment pathway incorporating whole-breast irradiation [4]. Consequently, the current findings should not be extrapolated to the simultaneous omission of both SLNB and breast irradiation. Preoperative eligibility and the treatment scenario generated by the virtual tumor board should be distinguished. For patients younger than 65 years, the ASCO SLNB-omission pathway incorporates whole-breast irradiation; eligibility for partial-breast irradiation under separate radiotherapy recommendations does not establish suitability for combining that approach with SLNB omission [1,13]. The age-stratified transition matrices and the additional sensitivity analysis clarify the contribution of recommendations outside the whole-breast irradiation scenario to the observed decision-change rate.

The limited effect on chemotherapy was consistent with the increasing primacy of tumor biology over anatomical nodal stage in postmenopausal hormone receptor-positive/HER2-negative disease. Although the genomic-testing category changed in 32 patients, this should not be interpreted as 32 newly requested assays: testing was newly recommended in 15 patients and withdrawn in 10. Definitive chemotherapy recommendations increased from six to 13 patients, corresponding to only seven management changes (1.7%). Kalinsky et al. demonstrated that postmenopausal patients with one to three positive nodes and a 21-gene recurrence score ≤ 25 did not derive a significant chemotherapy benefit, providing a biological explanation for the low chemotherapy impact observed in our cohort [5]. Andre et al. similarly emphasized the role of validated genomic assays in guiding chemotherapy decisions in postmenopausal hormone receptor-positive/HER2-negative disease [14]. The sensitivity analysis in which existing genomic results were displayed at both assessment stages produced a similar decision-change rate of 6.1%, suggesting that the main finding was not driven exclusively by concealment of genomic information.

In contrast, nodal disclosure had a greater effect on CDK4/6-inhibitor recommendations, which increased from two to 15 patients. Wanis et al. estimated that nodal information would affect chemotherapy eligibility in only 0.4% of postmenopausal patients but could affect abemaciclib and ribociclib eligibility in 2.1% and 7.5%, respectively [7]. Slamon et al. demonstrated improved invasive disease-free survival with adjuvant ribociclib in eligible patients with stage II–III hormone receptor-positive/HER2-negative breast cancer [6]. Heidinger et al. subsequently estimated node-dependent eligibility rates of 3.2% for abemaciclib under regulatory criteria and 7.8% for ribociclib among 309 patients with small luminal tumors [24]. The 3.2% increase in multidisciplinary CDK4/6-inhibitor recommendations should be interpreted in relation to the distinct eligibility frameworks for the individual agents. Under the monarchE cohort 1 definition, limited nodal involvement requires additional high-risk features to support abemaciclib eligibility, whereas the NATALEE framework encompasses a broader anatomical stage II–III population [6,16,17]. Nevertheless, isolated tumor cells and pT1pN1mi disease should not be equated with macrometastatic N1 disease when evaluating node-dependent ribociclib eligibility [18]. Furthermore, meeting clinicopathological eligibility criteria does not establish that treatment offers a favorable benefit–risk balance for every patient. The observed changes therefore reflect individualized multidisciplinary recommendations rather than automatic treatment assignment or demonstrated benefit within this cohort.

Disclosure of SLNB pathology resulted in active additional axillary treatment recommendations in only 10 patients (2.4%), including eight recommendations for axillary radiotherapy and two for completion axillary dissection. Giuliano et al. demonstrated that completion axillary dissection could be omitted without compromising 10-year overall survival in appropriately selected patients with one or two positive sentinel nodes undergoing breast-conserving treatment [25]. Bartels et al. reported excellent long-term axillary control with both axillary radiotherapy and axillary dissection in the AMAROS trial, with less lymphedema after radiotherapy [26]. De Boniface et al. further demonstrated in SENOMAC that omission of completion axillary dissection was noninferior in clinically node-negative patients with one or two sentinel-node macrometastases [27]. The low rate of additional axillary-treatment recommendations in our study is therefore consistent with contemporary axillary de-escalation. Nodal positivity alone should not be assumed to require completion dissection or axillary irradiation; such recommendations should be linked to the extent of nodal burden, extranodal extension, and other relevant risk factors.

Nodal disclosure also increased decision confidence and agreement, particularly for radiotherapy and CDK4/6 inhibition. The median case-level confidence score increased by 0.50 points, and consensus without adjudication increased from 94.4% to 97.1%. These results indicate that pathological nodal information reduces multidisciplinary uncertainty even when it does not change the final recommendation. However, greater clinician confidence is not a patient-centered outcome and does not establish that SLNB is clinically necessary. Reimer et al. demonstrated that SLNB was associated with greater arm and breast symptoms than omission of axillary surgery, with clinically relevant differences in arm symptoms persisting for up to 18 months [2]. The informational yield observed in our study must therefore be balanced against procedure-related morbidity and the potential adverse effects of additional treatment prompted by nodal disclosure.

The principal strengths of this study include the denominator-based analysis of the entire eligible cohort, assessment of multiple systemic and radiotherapy domains, and the paired evaluation of identical patients before and after disclosure of nodal pathology. Locking first-stage decisions, recoding and re-randomizing cases, and applying a washout period reduced—but could not eliminate—recall and anchoring effects. The conservative primary endpoint distinguished definitive treatment changes from changes in testing or uncertainty, while the expanded endpoint captured broader informational consequences. The stability of the results after restriction to the postoperatively confirmed low-risk cohort, alternative classification of isolated tumor cells, and disclosure of available genomic results supports the internal robustness of the principal estimate.

Several limitations should nevertheless be acknowledged. First, this was a retrospective, single-center study restricted to patients who had undergone successful SLNB and had sufficiently complete records, creating potential selection and information bias and limiting generalizability to institutions with different systemic-treatment or radiotherapy practices. Second, the tumor board generated counterfactual recommendations rather than observing actual decisions prospectively; patient preferences, treatment access, toxicity considerations, and shared decision-making could not be fully reproduced. No inference can therefore be made regarding recurrence, survival, quality of life, or the net clinical benefit of the management changes. Third, the node-concealed assessment included definitive breast pathology and consequently measured the incremental postoperative value of nodal information, rather than the accuracy of preoperative selection for SLNB omission. Fourth, the same specialists participated in both stages. Despite the washout period and recoding, residual recall, learning, and test–retest variability remain possible; the two radiotherapy de-escalations among pN0 patients may partly reflect such variability. Fifth, cases treated between 2015 and 2026 were reassessed according to contemporary standards, although genomic testing, CDK4/6-inhibitor availability, and radiotherapy practice evolved during this period. The similar decision-change rates across treatment eras are reassuring but do not establish equivalence. Sixth, the composite endpoint assigned equal weight to changes of potentially different clinical magnitude, and the exploratory secondary analyses were not adjusted for multiple comparisons. Finally, the limited number of primary events constrained model complexity. Firth regression reduced small-sample bias but did not convert the model into a validated prediction tool, and external validation is required before any pre-SLNB factor is used for patient selection.

## 5. Conclusions

In conclusion, sentinel-node pathology left definitive adjuvant-treatment recommendations unchanged in more than 90% of postmenopausal patients meeting the study-defined clinicopathological and imaging criteria derived from the 2025 ASCO omission pathway. Nevertheless, nodal information produced a clinically meaningful recommendation change in approximately one in 15 patients, predominantly through radiotherapy-field selection and CDK4/6-inhibitor consideration rather than conventional chemotherapy. Management-changing information was strongly concentrated among patients with micrometastatic or macrometastatic disease and was more frequent with increasing clinical tumor size. These findings support selective, multidisciplinary implementation of SLNB omission rather than universal omission or routine staging. Multicenter prospective studies using independent panels and linking recommendation changes to actual treatment, patient-reported outcomes, recurrence, and survival are needed to determine whether the incremental information provided by SLNB translates into net clinical benefit.

## Figures and Tables

**Figure 1:**
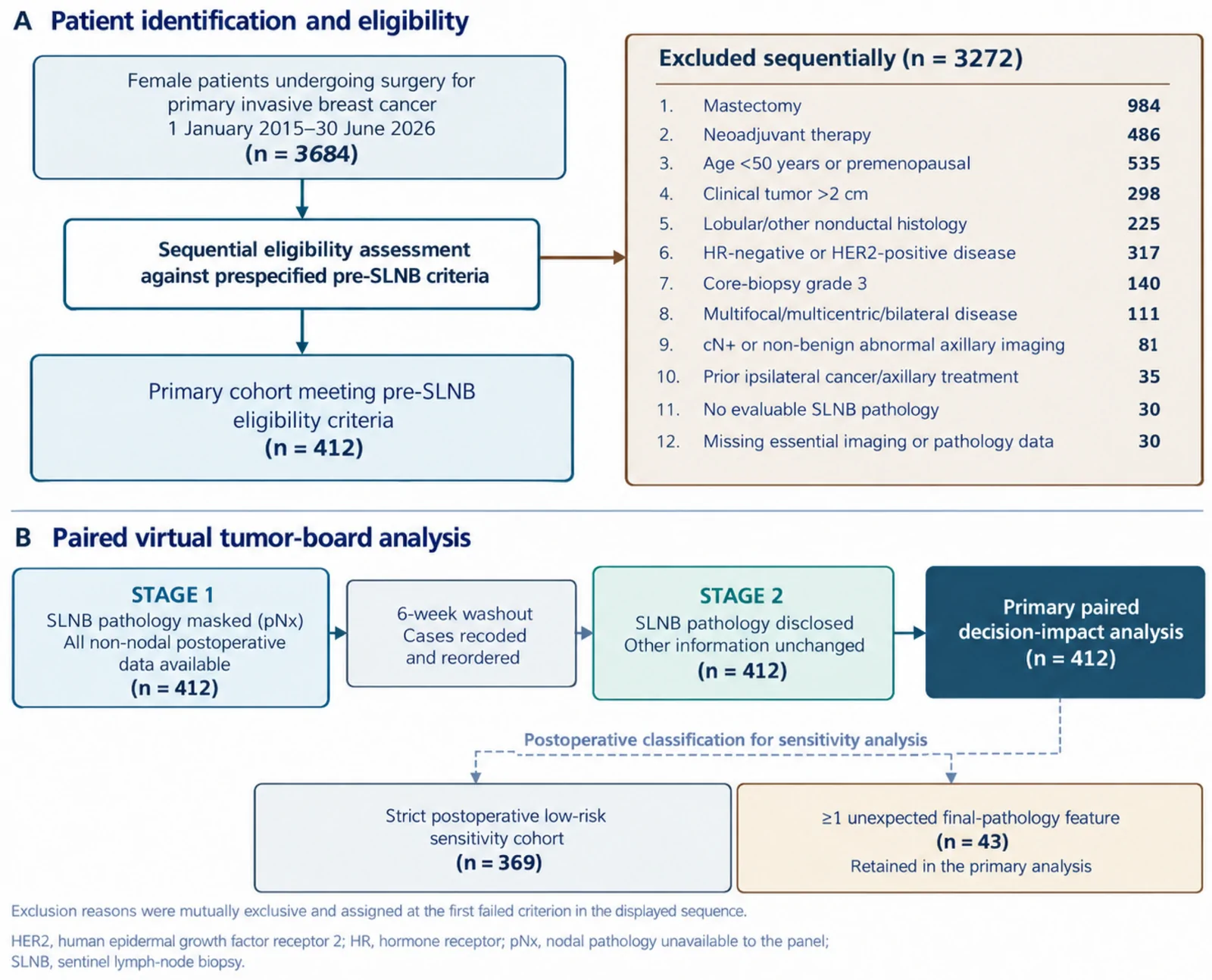
Flow diagram of patient identification, sequential eligibility assessment, and inclusion in the paired virtual tumor-board analysis.

**Figure 2:**
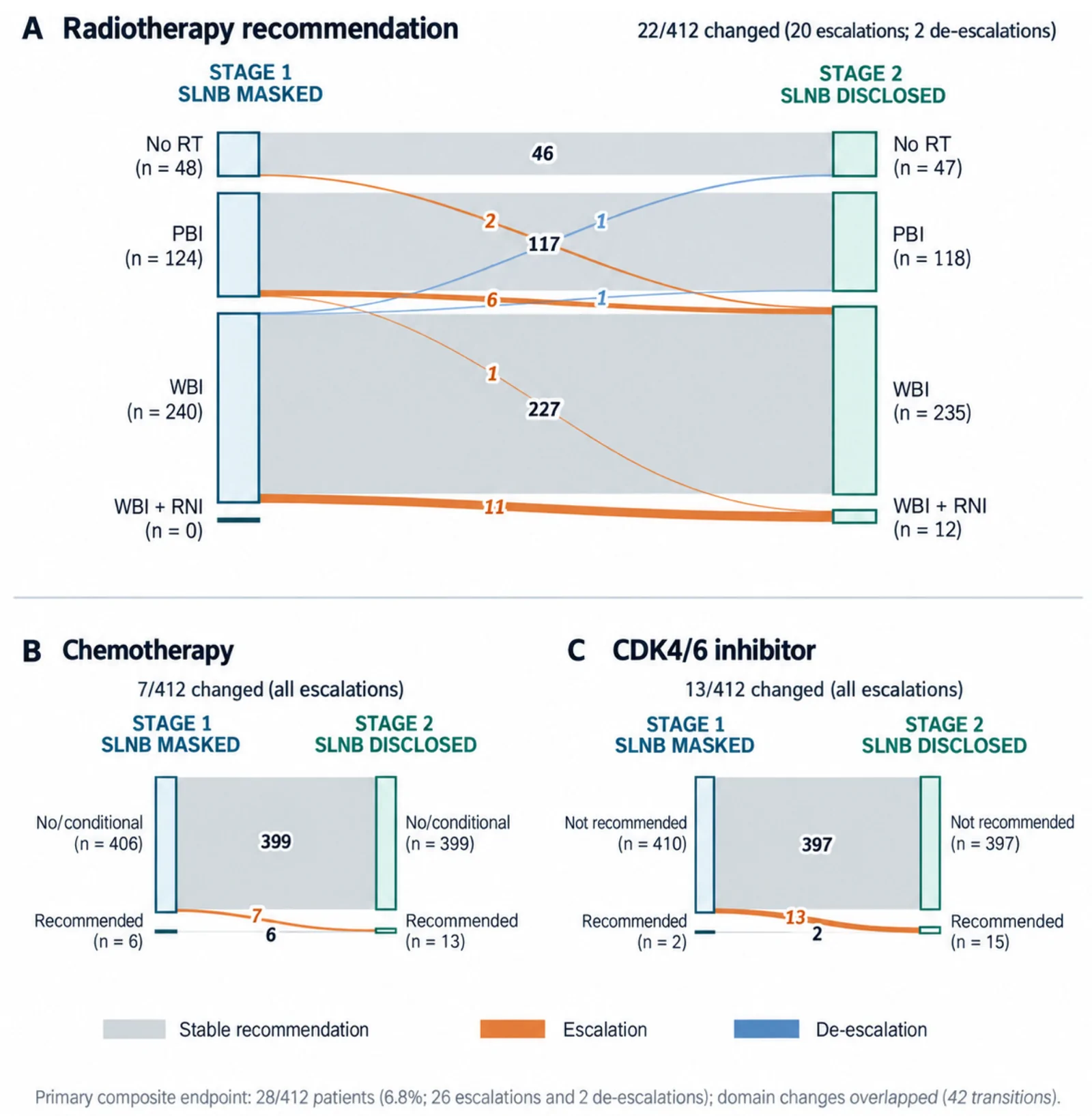
Paired transitions in multidisciplinary treatment recommendations before and after disclosure of sentinel lymph-node pathology.

**Figure 3:**
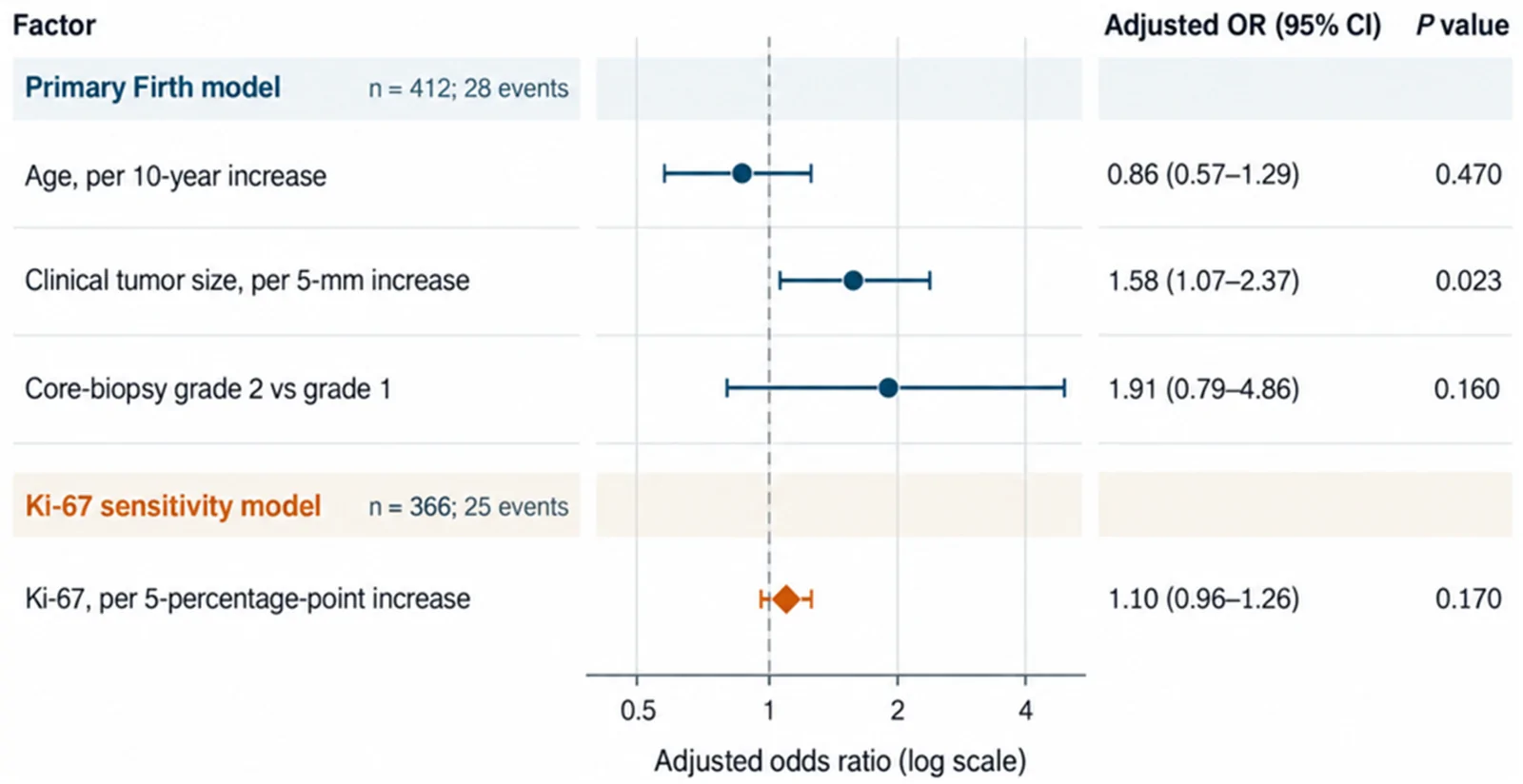
Pre-SLNB factors associated with management-changing sentinel-node information. Forest plot of adjusted odds ratios and 95% profile-likelihood confidence intervals from the Firth penalized logistic regression model. The primary model included age, clinical tumor size, and the core-biopsy grade and was fitted in 412 patients with 28 management-changing events. Ki-67 was evaluated separately in a complete-case sensitivity model including 366 patients and 25 events, with adjustment for the variables in the primary model.

**Table 1:** Baseline clinicopathological characteristics according to sentinel lymph-node tumor-deposit status.

Variable	Overall (*N* = 412)	No Nodal Deposit (*n* = 366)	Any Nodal Deposit (*n* = 46)	*p* Value
Age, years	65.0 ± 7.7	65.2 ± 7.8	63.7 ± 7.1	0.205
Age 50–64 years	197 (47.8)	171 (46.7)	26 (56.5)	0.215
Age ≥ 65 years	215 (52.2)	195 (53.3)	20 (43.5)	
Body mass index, kg/m^2^	28.3 ± 4.6	28.2 ± 4.5	28.8 ± 5.0	0.382
Clinical tumor size, mm	14 (10–17)	13 (10–16)	16 (12–19)	0.004
Clinical tumor size ≤ 10 mm	158 (38.3)	148 (40.4)	10 (21.7)	0.015
Clinical tumor size 11–20 mm	254 (61.7)	218 (59.6)	36 (78.3)	
Core-biopsy grade 1	151 (36.7)	142 (38.8)	9 (19.6)	0.014
Core-biopsy grade 2	261 (63.3)	224 (61.2)	37 (80.4)	
ER-low positive, 1–10%	13 (3.2)	12 (3.3)	1 (2.2)	>0.999
Progesterone receptor expression <20%	78 (18.9)	66 (18.0)	12 (26.1)	0.229
Ki-67 proliferation index, %	14 (8–21)	13 (8–20)	18 (12–25)	0.003
Completely normal axillary ultrasonography	392 (95.1)	350 (95.6)	42 (91.3)	0.261
One suspicious node with benign concordant biopsy	20 (4.9)	16 (4.4)	4 (8.7)	
Standard breast-conserving surgery	267 (64.8)	238 (65.0)	29 (63.0)	0.870
Oncoplastic breast-conserving surgery	145 (35.2)	128 (35.0)	17 (37.0)	
Definitive pathological tumor size, mm	15 (11–18)	14 (10–18)	17 (13–20)	<0.001
Unexpected pathological tumor size > 20 mm	18 (4.4)	12 (3.3)	6 (13.0)	0.009
Definitive pathological grade 1	135 (32.8)	126 (34.4)	9 (19.6)	0.012
Definitive pathological grade 2	260 (63.1)	228 (62.3)	32 (69.6)	
Definitive pathological grade 3	17 (4.1)	12 (3.3)	5 (10.9)	
Lymphovascular invasion	34 (8.3)	22 (6.0)	12 (26.1)	<0.001
Unexpected multifocality	15 (3.6)	11 (3.0)	4 (8.7)	0.074
Unexpected nonductal invasive component	6 (1.5)	5 (1.4)	1 (2.2)	0.511
At least one unexpected postoperative pathological feature	43 (10.4)	30 (8.2)	13 (28.3)	<0.001

Data are presented as means ± standard deviations, medians (interquartile ranges), or *n* (%). Isolated tumor cells are included in the “any nodal deposit” group. ER, estrogen receptor.

**Table 2:** Sentinel lymph-node biopsy and pathological nodal findings.

Variable	Value
Sentinel nodes removed, median (IQR)	2 (1–3)
Sentinel nodes removed, range	1–7
One sentinel node removed	132 (32.0)
Two sentinel nodes removed	154 (37.4)
Three or more sentinel nodes removed	126 (30.6)
No nodal tumor deposit, pN0(i−)	366 (88.8)
Any nodal tumor deposit	46 (11.2)
Isolated tumor cells, pN0(i+)	7 (1.7)
Micrometastasis, pN1mi	13 (3.2)
Macrometastatic sentinel-node disease	26 (6.3)
Largest metastatic deposit, mm *	2.6 (0.7–6.4)
Largest deposit ≤0.2 mm *	7 (15.2)
Largest deposit >0.2–2.0 mm *	13 (28.3)
Largest deposit >2–5 mm *	12 (26.1)
Largest deposit >5 mm *	14 (30.4)
One involved sentinel node *	38 (82.6)
Two involved sentinel nodes *	6 (13.0)
Three involved sentinel nodes *	2 (4.3)
Sentinel-node ratio *	0.50 (0.33–1.00)
Extranodal extension *	6 (13.0)
Extranodal extension ≤2 mm *	4 (8.7)
Extranodal extension >2 mm *	2 (4.3)

Data are *n* (%) or median (interquartile range). Percentages without an asterisk use the entire cohort as the denominator. * Denominator: patients with any nodal tumor deposit (*n* = 46).

**Table 3:** Paired comparison of multidisciplinary recommendations before and after disclosure of sentinel lymph-node pathology.

Recommendation	Stage 1: Node Concealed, *n* (%)	Stage 2: Node Disclosed, *n* (%)	Newly Recommended	Withdrawn	*p* Value
Genomic assay recommended	96 (23.3)	101 (24.5)	15	10	0.424
Cytotoxic chemotherapy recommended	6 (1.5)	13 (3.2)	7	0	0.016
CDK4/6 inhibitor recommended	2 (0.5)	15 (3.6)	13	0	<0.001
No radiotherapy	48 (11.7)	47 (11.4)	—	—	
Partial-breast irradiation	124 (30.1)	118 (28.6)	—	—	
Whole-breast irradiation	240 (58.3)	235 (57.0)	—	—	
Whole-breast irradiation plus RNI	0 (0.0)	12 (2.9)	12	0	0.001 *
Active additional axillary treatment	0 (0.0)	10 (2.4)	10	0	0.002
Any clinically meaningful primary change	—	28 (6.8)	—	—	—
Expanded decision-impact endpoint	—	43 (10.4)	—	—	—

Stage-specific percentages use the entire cohort (*N* = 412). For chemotherapy and CDK4/6-inhibitor therapy, “newly recommended” and “withdrawn” denote definitive No → Yes and Yes → No transitions, respectively; exact McNemar comparisons were restricted to patients with definitive recommendations at both stages. Conditional and indeterminate transitions are reported separately in Supplementary Table S2A and did not independently constitute primary events. For genomic testing, “newly recommended” includes all transitions into the recommended category, and “withdrawn” includes all transitions out of that category, including transitions involving indeterminate recommendations. Treatment domains overlap; primary and expanded endpoint totals count each patient once. * *p* value for the overall radiotherapy-category transition was calculated using the Stuart–Maxwell test. RNI, regional nodal irradiation.

**Table 4:** Transition matrix of radiotherapy recommendations between the node-concealed and node-disclosed assessments.

Stage 1 Recommendation	Stage 2: No RT	Stage 2: PBI	Stage 2: WBI	Stage 2: WBI + RNI	Total
No radiotherapy	46	0	2	0	48
Partial-breast irradiation	0	117	6	1	124
Whole-breast irradiation	1	1	227	11	240
Whole-breast irradiation + RNI	0	0	0	0	0
**Total**	47	118	235	12	412

Values on the diagonal represent stable recommendations; off-diagonal values represent changes following nodal disclosure. Twenty changes represented escalation and two represented de-escalation. Stuart–Maxwell χ^2^ = 15.49, 3 degrees of freedom, *p* = 0.001. PBI, partial-breast irradiation; RT, radiotherapy; RNI, regional nodal irradiation; WBI, whole-breast irradiation.

**Table 5:** Firth penalized logistic regression analysis of pre-SLNB factors associated with management-changing nodal information.

Pre-SLNB Variable	Events/Total, *n*/*N*	Unadjusted OR (95% CI)	*p* Value	Adjusted OR (95% CI)	*p* Value
Age, per 10-year increase	28/412	0.78 (0.56–1.09)	0.142	0.86 (0.57–1.29)	0.470
Clinical tumor size, per 5 mm increase	28/412	1.72 (1.23–2.46)	0.002	1.58 (1.07–2.37)	0.023
Core-biopsy grade 1	5/151	Reference	—	Reference	—
Core-biopsy grade 2	23/261	2.74 (1.08–7.92)	0.033	1.91 (0.79–4.86)	0.160
Ki-67, per 5-percentage-point increase *	25/366	1.16 (1.04–1.30)	0.010	1.10 (0.96–1.26)	0.170

The primary adjusted model included age, clinical tumor size, and core-biopsy grade. * Ki-67 was added only in the complete-case sensitivity model. Confidence intervals are profile-likelihood intervals. OR, odds ratio; SLNB, sentinel lymph-node biopsy.

**Table 6:** Patterns of clinically meaningful decision changes and their relationship with sentinel-node disease burden.

A. Pattern of Decision Changes		
Outcome		*n* (%)
Any clinically meaningful primary change		28 (6.8)
Escalation only		26 (6.3)
De-escalation only		2 (0.5)
One primary treatment domain changed		17 (4.1)
Two primary treatment domains changed		8 (1.9)
Three primary treatment domains changed		3 (0.7)
Radiotherapy category changed		22 (5.3)
Chemotherapy recommendation changed		7 (1.7)
CDK4/6-inhibitor recommendation changed		13 (3.2)
Genomic-testing category changed *		32 (7.8)
Active additional axillary treatment recommended *		10 (2.4)
Additional axillary evaluation recommended *		3 (0.7)
Expanded decision-impact endpoint *		43 (10.4)
**B. Primary outcome according to SLNB category**		
**SLNB category**	**Total *n***	**Clinically meaningful change, *n* (%)**
pN0	366	2 (0.5)
pN0(i+)	7	0 (0.0)
pN1mi	13	6 (46.2)
Macrometastatic disease	26	20 (76.9)
Any nodal tumor deposit	46	26 (56.5)
**Entire cohort**	412	28 (6.8)

Categories marked with an asterisk were secondary outcomes and were not included in the conservative primary endpoint unless accompanied by a definitive systemic treatment or radiotherapy change. Overall comparison across mutually exclusive pN categories: *p* < 0.001.

**Table 7:** Interobserver agreement and decision confidence before and after disclosure of SLNB pathology.

Decision Domain	Stage 1 Agreement, %	Stage 1 Fleiss κ (95% CI)	Stage 2 Agreement, %	Stage 2 Fleiss κ (95% CI)
Genomic testing	82.8	0.58 (0.53–0.63)	89.9	0.67 (0.62–0.72)
Chemotherapy	91.5	0.69 (0.64–0.74)	94.8	0.76 (0.71–0.81)
CDK4/6 inhibition	87.9	0.55 (0.49–0.61)	93.2	0.70 (0.65–0.75)
Radiotherapy category	77.4	0.52 (0.48–0.56)	85.6	0.65 (0.61–0.69)
Further axillary management	96.8	Not estimable *	95.6	0.74 (0.68–0.80)
Decision confidence, median (IQR)	3.7 (3.3–4.0)	—	4.2 (3.8–4.5)	—

* κ was unstable because “no additional axillary management” was selected almost universally during the node-concealed stage. Confidence was scored from 1 to 5, with higher values indicating greater confidence. CI, confidence interval; SLNB, sentinel lymph-node biopsy.

## Data Availability

The original contributions presented in this study are included in the article. Further inquiries can be directed to the corresponding author.

## Supplementary Materials

The following supporting information can be downloaded at: https://www.mdpi.com/article/10.3390/diagnostics16213636/s1. Text S1. Treatment-specific decision framework for the paired virtual tumor-board assessments. Table S1. Patient-level clinicopathological and nodal findings, paired adjuvant-treatment recommendations, and documented reasons for management changes following disclosure of sentinel-node pathology in the 28 patients reaching the primary endpoint. Table S2A. The transition rules for primary and expanded decision-impact endpoints. Table S2B. The paired transitions in genomic-testing recommendation categories (*N* = 412). Table S2C. The mutually exclusive composition of the expanded decision-impact endpoint. Table S3. Age-stratified radiotherapy recommendations, paired transitions, and sensitivity analysis addressing departures from the whole-breast irradiation treatment scenario. Table S4. Availability of existing genomic-assay results and comparison of paired treatment recommendations under concealed and available genomic-information conditions.
