# Supplementary material for: Who Can Truly Skip the Sentinel Node? A Paired Two-Stage Virtual Tumor-Board Study Among Patients Meeting the 2025 ASCO Clinicopathologic Criteria
Source: Diagnostics (Basel). 2026 Nov 4;16(21):3636. doi: 10.3390/diagnostics16213636 (PMC13762201; doi:10.3390/diagnostics16213636)
Supplement: Supplementary file 1 [file diagnostics-16-03636-s001.zip › diagnostics-4603892-supplementary.pdf]

**Supplementary Text S1.** Treatment-specific decision framework for the paired virtual tumor-board assessments**.**

**1. Scope and information available during assessment**

The virtual tumor-board assessments evaluated the incremental contribution of sentinel lymph-node biopsy (SLNB) findings to contemporary adjuvant-treatment recommendations. The assessments were conducted for research purposes and did not influence the treatment received by individual patients. All cases were reviewed according to treatment standards applicable at the time of the virtual assessment, irrespective of the year of the original operation.

During Stage 1, the board received the clinical, imaging, core-biopsy, surgical, and definitive breast-pathology information available independently of SLNB. Pathological nodal status was designated pNx. Neither pathological node negativity nor node positivity was assumed. During Stage 2, the same nonnodal information was presented together with the number of sentinel nodes examined and involved, the category and size of the largest metastatic deposit, and the presence and extent of extranodal extension. Results of subsequent completion axillary dissection remained concealed. Consequently, differences between the two assessments reflected recommendations made with and without sentinel-node information against an otherwise unchanged clinical background. Existing genomic-assay results were concealed during both stages of the primary assessment. In the corresponding sensitivity analysis, available genomic results were disclosed identically at both stages.

**2. Recommendation categories and clinical interpretation**

Chemotherapy recommendations were categorized as recommended, not recommended, or conditional upon genomic testing. Genomic testing was recorded separately as not required, recommended, or indeterminate. CDK4/6-inhibitor recommendations were categorized as recommended, not recommended, or indeterminate. Radiotherapy recommendations were classified as omission, partial-breast irradiation (PBI), whole-breast irradiation (WBI), or WBI with regional nodal irradiation (RNI).

Formal treatment eligibility was distinguished from an individualized recommendation. Meeting an eligibility criterion did not automatically result in a recommendation for treatment. The board considered the applicable evidence together with comorbidity, anticipated toxicity, and expected absolute benefit.

**3. Chemotherapy and genomic testing**

Chemotherapy assessment incorporated menopausal status, hormone receptor and HER2 status, definitive tumor characteristics, nodal findings, and the potential contribution of genomic testing. Sentinel-node involvement was not interpreted as sufficient evidence of chemotherapy benefit in every postmenopausal patient with hormone receptor-positive/HER2-negative disease.

The clinical interpretation was informed by RxPONDER, in which postmenopausal patients with one to three positive nodes and a 21-gene recurrence score ≤25 did not obtain a significant benefit from adding chemotherapy to endocrine therapy [5]. ASCO guidance supported the use of validated genomic assays to inform chemotherapy decisions in appropriately selected postmenopausal patients with zero to three positive nodes [14]. Because genomic results were concealed in the primary assessment, patients were not assigned an assumed recurrence score or genomic-risk category. When genomic testing was required before a definitive decision could be reached, the chemotherapy recommendation remained conditional.

A transition from "await genomic testing" to "recommend chemotherapy" was distinguished from a transition from "chemotherapy not recommended" to "chemotherapy recommended." These transitions represented different starting levels of treatment certainty. A definitive recommendation made without a genomic result was interpreted according to its case-specific clinicopathological rationale, rather than as evidence that an unobserved genomic threshold had been met.

**4. Abemaciclib eligibility**

The study definition of abemaciclib eligibility followed the monarchE cohort 1 framework. Eligible patients had hormone receptor-positive/HER2-negative, node-positive disease with either:

- At least four positive axillary lymph nodes; or
- One to three positive axillary lymph nodes accompanied by histological grade 3 disease and/or a primary tumor measuring ≥50 mm.

Ki-67 ≥20% alone was not considered sufficient under this cohort 1-based definition [16].

Nodal criteria were evaluated using only the information available during the relevant assessment stage. Positive nodes identified through a subsequent completion axillary dissection were not added to the sentinel-node count for this assessment. The general ≥4-node criterion therefore did not authorize inference of additional involved nodes beyond those disclosed by SLNB. Eligibility was considered separately from the final recommendation, which incorporated the individual clinical context and anticipated benefit–risk balance.

**5. Ribociclib eligibility**

Ribociclib eligibility followed the NATALEE framework using AJCC eighth-edition anatomical stage rather than prognostic stage. The eligible categories comprised stage IIA disease with qualifying N1 involvement, stage IIB disease, and stage III disease [6,17].

For stage IIA node-negative disease, eligibility required histological grade 3 disease or grade 2 disease accompanied by Ki-67 ≥20% or a qualifying high genomic-risk result, such as an Oncotype DX recurrence score ≥26 [17]. A genomic-risk criterion requiring a concealed or unavailable assay result was not assumed to have been satisfied. Isolated tumor cells and pT1pN1mi disease were not considered sufficient, by themselves, to establish eligibility through the node-positive pathway [18]. Micrometastatic involvement was therefore distinguished from macrometastatic N1 disease rather than treating all sentinel-node deposits as interchangeable evidence of eligibility. As with abemaciclib, anatomical eligibility did not automatically produce a recommendation for ribociclib. The final recommendation incorporated comorbidity, anticipated toxicity, and expected absolute benefit.

**6. Selection of breast irradiation**

The indication for breast irradiation and the choice of breast target volume were assessed separately from the addition of RNI. Age, definitive breast pathology, and the remaining clinical context informed the assessment. Suitability for PBI was evaluated according to contemporary recommendations. Disclosure of micrometastatic or macrometastatic sentinel-node disease could support replacement of PBI with WBI because standard PBI recommendations did not include patients with positive lymph nodes [13]. This change was distinguished from a separate decision to add regional nodal treatment. The absence of pathological nodal information during Stage 1 was not interpreted as proof of node negativity. Similarly, an indication for RNI was not inferred solely from the absence of SLNB information.

**7. Regional nodal irradiation**

RNI assessment incorporated the number of involved sentinel nodes, metastatic deposit size, and the presence and extent of extranodal extension. These findings were interpreted alongside definitive tumor size, histological grade, lymphovascular invasion, age, and comorbidity. Isolated tumor cells were not considered an indication for nodal irradiation. Micrometastatic disease alone did not mandate RNI; WBI without elective nodal irradiation remained an option when the remaining clinicopathological features were favorable.

For macrometastatic disease, the board assessed the potential value of RNI rather than applying an automatic escalation rule. Greater nodal burden, extranodal extension, lymphovascular invasion, grade 3 disease, and an unexpectedly larger definitive tumor size favored consideration of regional treatment. One or two involved sentinel nodes did not independently require RNI in every patient [1,15]. The clinical rationale for replacing PBI with WBI was distinguished from the rationale for adding RNI. Thus, loss of suitability for PBI did not automatically establish a requirement for nodal irradiation.

**8. Paired assessment and interpretation of changes**

Panel members initially recorded their recommendations independently. Multidisciplinary discussion subsequently established the consensus recommendation, with unresolved disagreements adjudicated as described in the Methods. Stage 1 recommendations were locked before nodal disclosure. After a six-week washout period, cases were recoded and presented in a newly randomized order for Stage 2 assessment. Panel members did not have access to their Stage 1 responses. Paired recommendations retained the distinction between definitive, conditional, and indeterminate categories. Multiple treatment-domain changes in the same patient represented one patient with management-changing information. Changes in recommendation were interpreted as measures of decision impact; they did not establish treatment receipt, improved oncological outcomes, or net clinical benefit.

**Supplementary Table S1.** Patient-level clinicopathological and nodal findings, paired adjuvant-treatment recommendations, and documented reasons for management changes following disclosure of sentinel-node pathology in the 28 patients reaching the primary endpoint.

| **Panel A.** Clinical and breast pathology features. | | | | | | | |
|---|---|---|---|---|---|---|---|
| **ID** | **Age years** | **cT / pT mm** | **Core / final grade** | **ER / PR %** | **Ki-67 %** | **LVI** | **Strict low risk** |
| S01 | 58 | 19 / 24 | 2 / 3 | 90 / 10 | 38 | Yes | No |
| S02 | 61 | 20 / 26 | 2 / 3 | 90 / 5 | 42 | Yes | No |
| S03 | 57 | 18 / 20 | 2 / 2 | 90 / 10 | 35 | Yes | Yes |
| S04 | 68 | 17 / 19 | 2 / 2 | 90 / 70 | 18 | No | Yes |
| S05 | 62 | 16 / 17 | 2 / 2 | 90 / 60 | 20 | Yes | Yes |
| S06 | 60 | 14 / 15 | 2 / 2 | 90 / 80 | 16 | Yes | Yes |
| S07 | 64 | 18 / 19 | 2 / 3 | 90 / 15 | 32 | Yes | No |
| S08 | 59 | 17 / 18 | 2 / 2 | 90 / 50 | 22 | Yes | Yes |
| S09 | 66 | 16 / 18 | 1 / 2 | 90 / 60 | 19 | Yes | Yes |
| S10 | 69 | 20 / 23 | 2 / 3 | 90 / 10 | 36 | No | No |
| S11 | 56 | 18 / 20 | 2 / 2 | 5 / 1 | 45 | Yes | Yes |
| S12 | 72 | 13 / 16 | 1 / 2 | 90 / 80 | NA | No | Yes |
| S13 | 70 | 12 / 14 | 2 / 2 | 90 / 70 | 14 | No | Yes |
| S14 | 67 | 14 / 16 | 2 / 2 | 90 / 75 | 15 | No | Yes |
| S15 | 73 | 13 / 15 | 2 / 2 | 90 / 90 | 10 | No | Yes |
| S16 | 63 | 15 / 17 | 1 / 2 | 90 / 65 | 17 | No | Yes |
| S17 | 65 | 17 / 19 | 2 / 2 | 90 / 60 | 19 | No | Yes |
| S18 | 71 | 16 / 18 | 2 / 2 | 90 / 80 | 16 | No | Yes |
| S19 | 60 | 20 / 28 | 2 / 2 | 90 / 5 | 40 | Yes | No |
| S20 | 62 | 19 / 27 | 2 / 2 | 90 / 5 | 34 | Yes | No |
| S21 | 59 | 17 / 18 | 2 / 2 | 90 / 70 | 20 | Yes | Yes |
| S22 | 67 | 20 / 23 | 2 / 3 | 90 / 20 | 30 | No | No |
| S23 | 68 | 12 / 14 | 2 / 2 | 90 / 90 | 12 | No | Yes |
| S24 | 71 | 10 / 12 | 1 / 1 | 90 / 95 | NA | No | Yes |
| S25 | 74 | 13 / 15 | 2 / 2 | 90 / 85 | 13 | No | Yes |
| S26 | 76 | 14 / 16 | 2 / 2 | 90 / 80 | 14 | No | Yes |
| S27 | 73 | 14 / 15 | 2 / 2 | 90 / 90 | 12 | No | Yes |
| S28 | 78 | 10 / 11 | 1 / 1 | 90 / 95 | NA | No | Yes |

| **Panel B.** Sentinel node findings and radiotherapy decisions. | | | | | | | |
|---|---|---|---|---|---|---|---|
| **ID** | **SLN + / examined** | **Nodal category** | **Deposit mm** | **ENE mm** | **Stage 1 RT** | **Stage 2 RT** | **RT reason** |
| S01 | 1 / 2 | Macro | 6.5 | 0.5 | WBI | WBI+RNI | R-A |
| S02 | 2 / 3 | Macro | 8.0 | 1.5 | WBI | WBI+RNI | R-A |
| S03 | 3 / 4 | Macro | 9.0 | 3.0 | WBI | WBI+RNI | R-B |

| **Panel B.** Sentinel node findings and radiotherapy decisions. | | | | | | | |
|---|---|---|---|---|---|---|---|
| **ID** | **SLN + / examined** | **Nodal category** | **Deposit mm** | **ENE mm** | **Stage 1 RT** | **Stage 2 RT** | **RT reason** |
| S04 | 2 / 3 | Macro | 4.5 | 0 | PBI | WBI+RNI | R-B |
| S05 | 1 / 2 | Macro | 6.5 | 1.0 | WBI | WBI+RNI | R-C |
| S06 | 1 / 3 | Macro | 3.5 | 0 | WBI | WBI+RNI | R-C |
| S07 | 1 / 2 | Macro | 4.8 | 0 | WBI | WBI+RNI | R-A |
| S08 | 2 / 4 | Macro | 5.6 | 0 | WBI | WBI+RNI | R-B |
| S09 | 1 / 2 | Macro | 7.0 | 0 | WBI | WBI+RNI | R-C |
| S10 | 1 / 2 | Macro | 2.7 | 0 | PBI | WBI | R-E |
| S11 | 3 / 4 | Macro | 8.5 | 2.5 | WBI | WBI | — |
| S12 | 1 / 3 | Macro | 4.0 | 0.8 | WBI | WBI+RNI | R-C |
| S13 | 1 / 2 | Macro | 2.5 | 0 | PBI | WBI | R-E |
| S14 | 1 / 2 | Macro | 5.0 | 0 | PBI | WBI | R-E |
| S15 | 1 / 3 | Macro | 3.2 | 0 | PBI | WBI | R-E |
| S16 | 1 / 2 | Macro | 4.6 | 0 | WBI | WBI | — |
| S17 | 1 / 2 | Macro | 6.0 | 0 | WBI | WBI | — |
| S18 | 1 / 3 | Macro | 6.8 | 0 | WBI | WBI | — |
| S19 | 2 / 4 | Macro | 4.2 | 0 | WBI | WBI | — |
| S20 | 2 / 3 | Macro | 7.5 | 0 | WBI | WBI | — |
| S21 | 2 / 3 | pN1mi | 1.8 | 0 | WBI | WBI+RNI | R-D |
| S22 | 1 / 2 | pN1mi | 1.6 | 0 | WBI | WBI+RNI | R-D |
| S23 | 1 / 2 | pN1mi | 1.2 | 0 | PBI | WBI | R-E |
| S24 | 1 / 3 | pN1mi | 0.9 | 0 | PBI | WBI | R-E |
| S25 | 1 / 2 | pN1mi | 1.4 | 0 | None | WBI | R-F |
| S26 | 1 / 2 | pN1mi | 0.6 | 0 | None | WBI | R-F |
| S27 | 0 / 2 | pN0 | — | 0 | WBI | PBI | R-G |
| S28 | 0 / 1 | pN0 | — | 0 | WBI | None | R-H |

| **Panel C.** Systemic treatment decisions and domain overlap. | | | | | | |
|---|---|---|---|---|---|---|
| **ID** | **Genomic test Stage 1 → 2** | **Chemotherapy Stage 1 → 2** | **Chemo reason** | **CDK4/6 Stage 1 → 2** | **Drug criterion** | **Changed domains** |
| S01 | NR → NR | No → Yes | C1 | No → Abema | A1 | R+C+D |
| S02 | NR → NR | No → Yes | C1 | No → Abema | A1 | R+C+D |
| S03 | NR → NR | No → Yes | C2 | No → Ribo | B1 | R+C+D |
| S04 | Rec → Rec | Await → Await | — | No → Ribo | B1 | R+D |
| S05 | Rec → Rec | Await → Await | — | No → Ribo | B1 | R+D |
| S06 | NR → NR | No → No | — | No → Ribo | B1 | R+D |
| S07 | Rec → Rec | Await → Await | — | No → Abema | A1 | R+D |
| S08 | Rec → Rec | Await → Await | — | No → Ribo | B1 | R+D |
| S09 | Rec → Rec | Await → Await | — | No → Ribo | B1 | R+D |

| Panel C. Systemic treatment decisions and domain overlap. | | | | | | |
|---|---|---|---|---|---|---|
| **ID** | **Genomic test Stage 1 → 2** | **Chemotherapy Stage 1 → 2** | **Chemo reason** | **CDK4/6 Stage 1 → 2** | **Drug criterion** | **Changed domains** |
| S10 | NR → NR | No → Yes | C1 | No → No | — | R+C |
| S11 | NR → NR | No → Yes | C3 | No → Ribo | B1 | C+D |
| S12 | NR → NR | No → No | — | No → No | — | R |
| S13 | NR → NR | No → No | — | No → No | — | R |
| S14 | NR → NR | No → No | — | No → No | — | R |
| S15 | NR → NR | No → No | — | No → No | — | R |
| S16 | Rec → Rec | Await → Await | — | No → Ribo | B1 | D |
| S17 | Rec → Rec | Await → Await | — | No → Ribo | B1 | D |
| S18 | Rec → Rec | Await → Await | — | No → Ribo | B1 | D |
| S19 | NR → NR | No → Yes | C4 | No → No | — | C |
| S20 | NR → NR | No → Yes | C4 | No → No | — | C |
| S21 | NR → NR | No → No | — | No → No | — | R |
| S22 | NR → NR | No → No | — | No → No | — | R |
| S23 | NR → NR | No → No | — | No → No | — | R |
| S24 | NR → NR | No → No | — | No → No | — | R |
| S25 | NR → NR | No → No | — | No → No | — | R |
| S26 | NR → NR | No → No | — | No → No | — | R |
| S27 | NR → NR | No → No | — | No → No | — | R |
| S28 | NR → NR | No → No | — | No → No | — | R |

ENE, extranodal extension; G, histological grade; LVI, lymphovascular invasion; PBI, partial-breast irradiation; RNI, regional nodal irradiation; SLN, sentinel lymph node; WBI, whole-breast irradiation. For Panel A: All cases are postmenopausal and HR-positive/HER2-negative, with preoperative cT ≤20 mm and core grade 1–2. For Panel B: Macro: macrometastatic disease; pN1mi: deposit >0.2–2.0 mm; ENE: extranodal extension; PBI: partial-breast irradiation; WBI: whole-breast irradiation; RNI: regional nodal irradiation. RT reason codes are explained after panel C. A dash indicates no applicable deposit or no domain change. RT transitions: WBI → WBI+RNI, n=11; PBI → WBI+RNI, n=1; PBI → WBI, n=6; None → WBI, n=2; WBI → PBI, n=1; WBI → None, n=1. For Panel C: NR: genomic testing not required; Rec: genomic testing recommended; Await: chemotherapy decision conditional on genomic testing; Abema: abemaciclib; Ribo: ribociclib. R, C, and D denote radiotherapy, chemotherapy, and CDK4/6 changes, respectively.

**Table S2A.** The transition rules for primary and expanded decision-impact endpoints.

| Decision domain | Stage 1 → Stage 2 | n | Primary rule | Expanded rule |
|---|---|---|---|---|
| Radiotherapy | Any change among None, PBI, WBI and WBI + RNI | 22 | Yes | Yes |
| Chemotherapy | No → Yes | 7 | Yes | Yes |
| Chemotherapy | Yes → No | 0 | Yes | Yes |
| Chemotherapy | Await genomic testing → Yes | 0 | No | Yes (U) |
| Chemotherapy | Await genomic testing → No | 9 | No | Yes (U) |
| Chemotherapy | No or Yes → Await genomic testing | 15 | No | No, by itself |
| CDK4/6 inhibitor | No → Yes | 13 | Yes | Yes |
| CDK4/6 inhibitor | Yes → No | 0 | Yes | Yes |
| CDK4/6 inhibitor | Indeterminate → Yes | 0 | No | Yes (U) |
| CDK4/6 inhibitor | Indeterminate → No | 5 | No | Yes (U) |
| CDK4/6 inhibitor | No or Yes → Indeterminate | 0 | No | No, by itself |
| Genomic testing | Any change among NR, Rec and Ind | 32 | No | Yes (G) |

NR, not required; Rec, recommended; Ind, indeterminate; PBI, partial-breast irradiation; WBI, whole-breast irradiation; RNI, regional nodal irradiation. Rules apply to the transition itself. A patient with a secondary transition can also satisfy P through another domain.

**Table S2B.** The paired transitions in genomic-testing recommendation categories (N = 412).

| Stage 1 category | Stage 2 Not required | Stage 2 Recommended | Stage 2 Indeterminate | Stage 1 total |
|---|---|---|---|---|
| Not required | 280 | 12 | 4 | 296 |
| Recommended | 8 | 86 | 2 | 96 |
| Indeterminate | 3 | 3 | 14 | 20 |
| Stage 2 total | 291 | 101 | 20 | 412 |

**Table S2C.** The mutually exclusive composition of the expanded decision-impact endpoint.

| P | G | U | n | Mutually exclusive patient group |
|---|---|---|---|---|
| Yes | No | No | 8 | Primary endpoint only |
| Yes | Yes | No | 13 | Primary change and genomic change |
| Yes | No | Yes | 0 | Primary change and resolution only |
| Yes | Yes | Yes | 7 | Primary change, genomic change and resolution |
| No | Yes | No | 8 | Additional patients with genomic change only |
| No | No | Yes | 3 | Additional patients with resolution only |
| No | Yes | Yes | 4 | Additional patients with both secondary changes |
| Total | | | 43 | Expanded endpoint: unique patients with P, G or U |

**Supplementary Table S3.** Age-stratified radiotherapy recommendations, paired transitions, and sensitivity analysis addressing departures from the whole-breast irradiation treatment scenario.

**S3A.** Radiotherapy recommendations by age and assessment stage, n (%).

| Recommendation | 50–64 Stage 1 (n = 197) | 50–64 Stage 2 (n = 197) | ≥65 Stage 1 (n = 215) | ≥65 Stage 2 (n = 215) | Overall Stage 1 (N = 412) | Overall Stage 2 (N = 412) |
|---|---|---|---|---|---|---|
| Omission | 0 (0.0) | 0 (0.0) | 48 (22.3) | 47 (21.9) | 48 (11.7) | 47 (11.4) |
| PBI | 12 (6.1) | 12 (6.1) | 112 (52.1) | 106 (49.3) | 124 (30.1) | 118 (28.6) |
| WBI | 185 (93.9) | 177 (89.8) | 55 (25.6) | 58 (27.0) | 240 (58.3) | 235 (57.0) |
| WBI + RNI | 0 (0.0) | 8 (4.1) | 0 (0.0) | 4 (1.9) | 0 (0.0) | 12 (2.9) |
| Total | 197 (100) | 197 (100) | 215 (100) | 215 (100) | 412 (100) | 412 (100) |

**S3B.** Complete paired radiotherapy transitions by age, n.

| Age 50–64 years (n = 197) | | | | | |
|---|---|---|---|---|---|
| **Stage 1 category** | **Stage 2 Omission** | **Stage 2 PBI** | **Stage 2 WBI** | **Stage 2 WBI + RNI** | **Stage 1 total** |
| Omission | 0 | 0 | 0 | 0 | 0 |
| PBI | 0 | 12 | 0 | 0 | 12 |
| WBI | 0 | 0 | 177 | 8 | 185 |
| WBI + RNI | 0 | 0 | 0 | 0 | 0 |
| Stage 2 total | 0 | 12 | 177 | 8 | 197 |
| Age ≥65 years (n = 215) | | | | | |
| **Stage 1 category** | **Stage 2 Omission** | **Stage 2 PBI** | **Stage 2 WBI** | **Stage 2 WBI + RNI** | **Stage 1 total** |
| Omission | 46 | 0 | 2 | 0 | 48 |
| PBI | 0 | 105 | 6 | 1 | 112 |
| WBI | 1 | 1 | 50 | 3 | 55 |
| WBI + RNI | 0 | 0 | 0 | 0 | 0 |
| Stage 2 total | 47 | 106 | 58 | 4 | 215 |

BI, partial-breast irradiation; WBI, whole-breast irradiation; RNI, regional nodal irradiation.

**S3C.** Primary endpoint before and after exclusion of younger patients with omission or PBI recommendations.

| Population | Excluded n | Analyzed N | Primary events, n | Rate, % | 95% Wilson CI, % |
|---|---|---|---|---|---|
| Full cohort | 0 | 412 | 28 | 6.8 | 4.7–9.6 |
| Age 50–64 years | 0 | 197 | 12 | 6.1 | 3.5–10.3 |
| Age ≥65 years | 0 | 215 | 16 | 7.4 | 4.6–11.7 |
| Sensitivity cohort after exclusion | 12 | 400 | 28 | 7.0 | 4.9–9.9 |
| Age 50–64 years after exclusion | 12 | 185 | 12 | 6.5 | 3.7–11.0 |

**S3D.** The younger-patient scenarios with PBI recommendations despite initial WBI intent.

| Case ID | Age, years | Tumour size / grade | Nodal status Stage 1 → 2 | RT Stage 1 → 2 | Primary event | Reason |
|---|---|---|---|---|---|---|
| S80 | 52 | 8 mm / G1 | pNx → pN0 | PBI → PBI | No | F1 |
| S81 | 54 | 9 mm / G1 | pNx → pN0 | PBI → PBI | No | F1 |
| S82 | 55 | 10 mm / G2 | pNx → pN0 | PBI → PBI | No | F1 |
| S83 | 56 | 7 mm / G1 | pNx → pN0 | PBI → PBI | No | F1 |
| S84 | 58 | 11 mm / G2 | pNx → pN0 | PBI → PBI | No | F1 |
| S85 | 59 | 9 mm / G1 | pNx → pN0 | PBI → PBI | No | F1 |
| S86 | 60 | 12 mm / G2 | pNx → pN0 | PBI → PBI | No | F1 |
| S87 | 61 | 10 mm / G1 | pNx → pN0 | PBI → PBI | No | F1 |
| S88 | 62 | 13 mm / G2 | pNx → pN0 | PBI → PBI | No | F1 |
| S89 | 63 | 8 mm / G1 | pNx → pN0 | PBI → PBI | No | F1 |
| S90 | 64 | 14 mm / G2 | pNx → pN0 | PBI → PBI | No | F1 |
| S91 | 64 | 15 mm / G2 | pNx → pN0 | PBI → PBI | No | F1 |

**Supplementary Table S4.** Availability of existing genomic-assay results and comparison of paired treatment recommendations under concealed and available genomic-information conditions.

**S4A.** Availability and distribution of existing genomic results.

| **Characteristic** | Value, IQR, interquartile range | **Denominator** |
|---|---|---|
| Interpretable result available | 104 (25.2%) | All 412 patients |
| No interpretable result available | 308 (74.8%) | All 412 patients |
| Assay platform: Oncotype DX | 104 (100%) | 104 assigned reports |
| Recurrence score 0–25 | 88 (84.6%) | 104 assigned reports |
| Recurrence score 26–100 | 16 (15.4%) | 104 assigned reports |
| Recurrence score, median (IQR) | 19 (15–23.25) | 104 assigned reports |
| Recurrence score, range | 8–40 | 104 assigned reports |

**S4B.** Chemotherapy recommendations under the two information conditions.

**B1 Stage-specific categories in the full cohort, n (%)**

| **Recommendation** | **Concealed, Stage 1** | **Concealed, Stage 2** | **Available, Stage 1** | **Available, Stage 2** |
|---|---|---|---|---|
| Recommend chemotherapy | 6 (1.5) | 13 (3.2) | 16 (3.9) | 20 (4.9) |
| Do not recommend chemotherapy | 309 (75.0) | 296 (71.8) | 387 (93.9) | 384 (93.2) |
| Await genomic testing | 97 (23.5) | 103 (25.0) | 9 (2.2) | 8 (1.9) |
| Total | 412 (100) | 412 (100) | 412 (100) | 412 (100) |
| Stage 1 category | Stage 2<br>No | Stage 2<br>Yes | Stage 2<br>Await | Stage 1<br>total |
| No | 287 | 7 | 15 | 309 |
| Yes | 0 | 6 | 0 | 6 |
| Await genomic testing | 9 | 0 | 88 | 97 |
| Stage 2 total | 296 | 13 | 103 | 412 |

**B2 Genomic results concealed at both nodal stages**

| **Stage 1 category** | **Stage 2, No** | **Stage 2, Yes** | **Stage 2, Await** | **Stage 1, total** |
|---|---|---|---|---|
| No | 375 | 4 | 8 | 387 |
| Yes | 0 | 16 | 0 | 16 |
| Await genomic testing | 9 | 0 | 0 | 9 |
| Stage 2 total | 384 | 20 | 8 | 412 |

**B3 Existing genomic results available at both nodal stages**

| **Stage 1 category** | **Stage 2, No** | **Stage 2, Yes** | **Stage 2, Await** | **Stage 1, total** |
|---|---|---|---|---|
| No | 375 | 4 | 8 | 387 |
| Yes | 0 | 16 | 0 | 16 |
| Await genomic testing | 9 | 0 | 0 | 9 |
| Stage 2 total | 384 | 20 | 8 | 412 |

| **S4C.** Patient-level reconciliation of the composite primary endpoint. | | | |
|---|---|---|---|
| **C1 Cross-classification across the two information conditions** | | | |
| **Primary endpoint with results concealed** | **Results available: Primary event** | **Results available: No primary event** | **Total** |
| Primary event | 25 | 3 | 28 |
| No primary event | 0 | 384 | 384 |
| Total | 25 | 387 | 412 |

| **C2 Primary endpoint by result availability** | | | | |
|---|---|---|---|---|
| **Population** | **Concealed n/N (%)** | **95% Wilson CI, %** | **Available n/N (%)** | **95% Wilson CI, %** |
| Full cohort | 28/412 (6.8%) | 4.7–9.6 | 25/412 (6.1%) | 4.1–8.8 |
| Existing result available | 10/104 (9.6%) | 5.3–16.8 | 7/104 (6.7%) | 3.3–13.2 |
| No existing result | 18/308 (5.8%) | 3.7–9.0 | 18/308 (5.8%) | 3.7–9.0 |
